# Antibody–drug conjugates in colorectal cancer: advances in targeted delivery and personalized oncology

**DOI:** 10.3389/fbioe.2026.1756741

**Published:** 2026-02-12

**Authors:** Huanle Wang, Xueyan Yan, Cong Xia, Danni Chen, Bo Pan, Yinan Zhang, Qianshi Zhang

**Affiliations:** 1 Department of Gastrointestinal Surgery, The Second Hospital of Dalian Medical University, Dalian, China; 2 Digestive System Disease Diagnosis and Treatment Center, The Second Hospital of Dalian Medical University, Dalian, China; 3 Ultrasound Department, The First Affiliated Hospital of Dalian Medical University, Dalian, China; 4 Department of Breast Surgery, The Second Hospital of Dalian Medical University, Dalian, China; 5 Department of Clinical Laboratory, The Second Hospital of Dalian Medical University, Dalian, China

**Keywords:** antibody–drug conjugates, colorectal cancer, drug delivery systems, multimodal cancer treatment, personalized medicine

## Abstract

Colorectal cancer remains among the most prevalent gastrointestinal malignancies worldwide, imposing a substantial clinical burden and highlighting the urgent need for precision and personalized treatment strategies. Conventional drug delivery approaches are limited by low selectivity, restricted bioavailability, and systemic toxicity, thereby limiting therapeutic efficacy. Antibody–drug conjugates, as advanced delivery plat-forms, have the advantages of highly specific antibody recognition, versatile cytotoxic payloads, and continuously evolving linker technologies. This combination provides novel opportunities to increase efficacy, reduce toxicity, and enable individualized precision therapy. Recent advances have demonstrated the potential of ADCs in CRC, yet challenges such as resistance, toxicity, and clinical translation persist. Multidisciplinary efforts among the pharmaceutical industry and molecular biology and clinical medicine fields will be essential to accelerate the development of more precise and personalized CRC therapies. This review summarizes the current research progress on ADCs as a treatment option for CRC, discusses innovations in delivery system design, examines the key challenges of personalization, and highlights future directions to better integrate ADCs into effective treatment paradigms.

## 1 Introduction

Colorectal cancer (CRC) is among the most common malignancies of the digestive system worldwide. It is the second leading cause of cancer-related mortality and ranks behind only lung and breast cancer in terms of incidence (Sung et al., 2021). Owing to the lack of specific early symptoms, most CRC patients are diagnosed at advanced stages, resulting in poor prognosis and limited therapeutic response (Wagle et al., 2025). The currently employed standard treatments are surgical resection, chemotherapy, radiotherapy, targeted agents, and immunotherapy. First-line chemotherapy drugs such as 5-fluorouracil and oxaliplatin are hindered by drug resistance, poor solubility and permeability, limited bioavailability, low specificity, and severe systemic side effects (Jeng et al., 2018). Radiotherapy can shrink tumours and relieve symptoms through precise localization, but tissue-specific sensitivity to radiation and adverse effects such as radiation enteritis remain limiting factors (Tan et al., 2022; Shi et al., 2023). Targeted therapies (Li Q. et al., 2024) and immunotherapies (Ganesh et al., 2019) have improved selectivity. However, their clinical utility is restricted by the rapid emergence of resistance mutations, the low response rate of “immune-cold” tumours, and immune-related toxicity.

CRC treatment strategies are strongly stage dependent; however, even patients with the same stage and treatment regimen show wide variability in survival outcomes. This discrepancy is primarily attributed to the biological heterogeneity of CRC, which manifests as profound molecular and spatiotemporal heterogeneity (Linnekamp et al., 2015; Jones et al., 2017; Suzuki et al., 2017). On the basis of gene expression profiling, consensus molecular subtypes (CMS1–4) have been defined, each of which exhibits distinct biological features and clinical behaviours (Guinney et al., 2015). This classification highlights CRC not as a single disease entity but as a spectrum of molecularly defined subgroups with divergent therapeutic sensitivities—posing fundamental challenges to the “one-size-fits-all” treatment paradigm. Spatiotemporal heterogeneity further influences therapeutic response and the development of treatment resistance (Marusyk et al., 2020). In parallel, the clinical demand for toxicity control underscores the necessity of shifting from systemic high-dose exposure to targeted delivery strategies (Han et al., 2024). Together, molecular heterogeneity, resistance, and toxicity management imperatives are driving the transition from uniform chemotherapy to highly personalized and targeted drug-delivery approaches. Against this backdrop, next-generation drug delivery systems—such as antibody–drug conjugates (ADCs)—offer promising solutions by enabling the selective targeting and localized release of potent cytotoxic agents (Markides et al., 2024).

ADCs, often described as “biomissiles” or “magic bullets,” are distinguished by their precision in drug delivery. The concept of “magic bullets” was originally proposed by the German immunologist Paul Ehrlich (Strebhardt and Ullrich, 2008), who envisioned agents that could directly reach their intended cellular targets without harming normal tissues. Over the past decade, advances in monoclonal antibody (mAb) engineering, antigen selection, linker chemistry, and payload design have led to substantial progress in ADC development (Fu et al., 2022; Tsuchikama et al., 2024). ADCs covalently couple mAbs with cytotoxic drugs through chemical linkers, combining the high specificity of antibody targeting with the potent tumour-killing capacity of small-molecule payloads (Tarantino et al., 2022). This dual advantage has established ADCs as a central focus in oncology drug development. As of August 2025, 17 ADCs have received regulatory approval worldwide (Table 1), representing a milestone in cancer therapeutics. Nevertheless, major challenges persist, including complex pharmacokinetics, toxicity, and the emergence of resistance. This review aims to discuss the development of ADCs for the treatment of CRC and explore how advanced delivery strategies may help overcome the barriers to personalized CRC therapy.

**TABLE 1:** Globally approved and marketed antibody‒drug conjugates.

Drug	Target	Cytotoxic payload	Average DAR	Developer/Holder company	First approval year	Approved indications
Mylotarg (gemtuzumab ozogamicin)	CD33	Calicheamicin	2–3	Pfizer	2000 (FDA)	Acute myeloid leukaemia
Adcetris (brentuximab vedotin)	CD30	MMAE	4	Seagen/Takeda	2011 (FDA)	Hodgkin lymphoma, systemic anaplastic large cell lymphoma, other T-cell lymphomas
Kadcyla (ado-trastuzumab emtansine, T-DM1)	HER2	DM1	3.5	Genentech/Roche	2013 (FDA)	HER2-positive breast cancer
Besponsa (inotuzumab ozogamicin)	CD22	Calicheamicin	6	Pfizer	2017 (FDA)	Relapsed/refractory B-cell precursor acute lymphoblastic leukaemia
Polivy (polatuzumab vedotin)	CD79b	MMAE	3.5–4	Genentech/Roche	2019 (FDA)	Relapsed/refractory diffuse large B-cell lymphoma
Padcev (enfortumab vedotin)	Nectin-4	MMAE	3.8	Astellas/Seagen	2019 (FDA)	Locally advanced or metastatic urothelial carcinoma
Enhertu (trastuzumab deruxtecan, T-DXd)	HER2	DXd	8	Daiichi Sankyo/AstraZeneca	2019 (FDA)	HER2-positive breast cancer, HER2-low breast cancer, gastric cancer, NSCLC, other HER2-expressing tumours
Trodelvy (sacituzumab govitecan)	Trop-2	SN-38	7.6	Immunomedics/Gilead Sciences	2020 (FDA)	Triple-negative breast cancer, urothelial cancer, HR+/HER2- breast cancer
Zynlonta (loncastuximab tesirine)	CD19	PBD dimer	2.3	ADC Therapeutics	2021 (FDA)	Relapsed/refractory diffuse large B-cell lymphoma
Tivdak (tisotumab vedotin)	Tissue Factor	MMAE	4	Genmab/Seagen	2021 (FDA)	Recurrent/metastatic cervical cancer
Elahere (mirvetuximab soravtansine)	Folate receptor-α	DM4	3.5–4	ImmunoGen	2022 (FDA)	Epithelial ovarian cancer/fallopian tube cancer/primary peritoneal carcinoma
Akalux (cetuximab sarotalocan)	EGFR	IR700 (photosensitizer)	2	Rakuten Medical	2020 (PMDA)	Head and neck cancer (photoimmunotherapy)
Aidixi (disitamab vedotin)	HER2	MMAE	3.5–4	RemeGen	2021 (NMPA)	Gastric, biliary tract, and breast cancer
Datroway (datopotamab deruxtecan)	TROP-2	DXd	8	Daiichi Sankyo/AstraZeneca	2024 (FDA)	NSCLC, breast cancer
Emrelis (telisotuzumab vedotin)	c-Met	MMAE	4	AbbVie	2025 (FDA)	NSCLC
Jiataile (sacituzumab tirumotecan)	TROP-2	Tirumotecan	6	TOT Biopharm	2025 (NMPA)	NSCLC (EGFR mutant)
Trastuzumab Rezetecan	HER2	Exatecan derivative	8	RemeGen	2025 (NMPA)	NSCLC (HER2 mutant)

Data cut-off December 2025. “First approval year” indicates the earliest regulatory approval (major agencies shown in parentheses). Subsequent key regulatory events are listed below:

Mylotarg (gemtuzumab ozogamicin): withdrawn in 2010 and reauthorized in 2017 (FDA).

Trodelvy (sacituzumab govitecan): additional approval in 2022 (NMPA).

## 2 ADC as an advanced drug delivery system

### 2.1 Composition and mechanism of action

**FIGURE 1:**
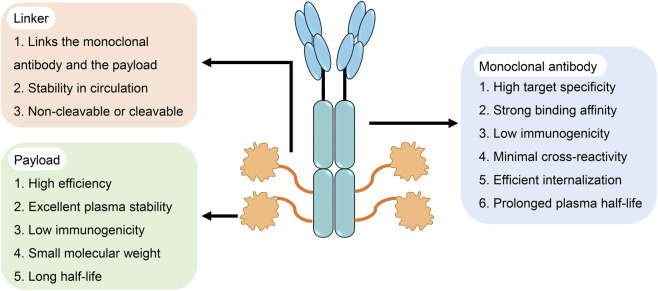
Structure and key properties of antibody–drug conjugates (ADCs). ADC comprises three essential components: a monoclonal antibody, a cytotoxic payload, and a linker.

An ADC is composed of three fundamental components: an antibody, a cytotoxic payload, and a linker (Figure 1). The antibody specifically binds to target antigens expressed on the surface of tumour cells and is internalized through clathrin-mediated endocytosis. Following internalization, the plasma membrane invaginates to form early endosomes, which subsequently mature into late endosomes and fuse with lysosomes, where proteolytic degradation occurs. This process results in the release of the cytotoxic payload, which exerts its effect by inducing DNA damage or disrupting microtubules, ultimately triggering apoptosis or cell death in tumour cells. In this way, ADCs achieve selective and potent antitumour activity by combining targeted recognition with efficient intracellular drug delivery.

In the early stages of ADC development, murine-derived antibodies were predominantly used. However, murine antibodies can act as antigens to trigger strong immune responses, leading to the activation, proliferation, and differentiation of immune cells, which often resulted in severe immune-related toxicity and contributed to the high failure rate of early ADCs (Hock et al., 2015; Kroemer et al., 2022). With the advent of recombinant technologies, chimeric and humanized antibodies progressively replaced murine antibodies, thereby substantially reducing immunogenicity (Abdollahpour-Alitappeh et al., 2019; Dyson et al., 2020). Currently, most ADCs are constructed using immunoglobulin G (IgG), the predominant immunoglobulin in human serum. IgG consists of four subclasses—IgG1, IgG2, IgG3, and IgG4 (Fu et al., 2022). Subtle structural differences among these subclasses can influence the solubility and half-life of monoclonal antibodies, as well as their binding affinities to various Fc gamma receptors expressed on immune effector cells (Sawant et al., 2020; Yu et al., 2020). Among these, IgG1 is the most abundant in circulation and has a relatively long serum half-life (approximately 21 days), as well as superior binding efficiency to Fc gamma receptors (Han et al., 2019). Importantly, IgG1 can elicit effector mechanisms such as antibody-dependent cellular cytotoxicity (ADCC), antibody-dependent cellular phagocytosis (ADCP), and complement-dependent cytotoxicity (CDC) (Natsume et al., 2009), making it the preferred subclass for ADC construction. The recombinant monoclonal antibody confers high specificity towards tumour-associated antigens, enabling the selective delivery of cytotoxic payloads to tumour sites—a critical initial step for ADCs to exert their antitumour effects (Dyson et al., 2020). The efficiency of ADC internalization also dictates therapeutic efficacy, which largely depends on the binding affinity between the antibody and its target antigen on the tumour cell surface. While stronger binding affinity may, in theory, accelerate internalization (Xu, 2015), the so-called “binding site barrier” in solid tumours can restrict tissue penetration (Tsumura et al., 2018; Singh et al., 2020). Therefore, an ADC monoclonal antibody should meet six criteria: high target specificity, strong binding affinity, low immunogenicity, minimal cross-reactivity, efficient internalization, and a prolonged plasma half-life (Khongorzul et al., 2020).

In ADCs, the cytotoxic payload is covalently conjugated to the antibody through a linker. An ideal linker should remain highly stable in plasma circulation but be readily cleaved upon internalization into tumour cells. Such dual properties are critical: maintaining stability during circulation prevents premature degradation and systemic release of the payload that can damage healthy tissues, while efficient cleavage ensures that the payload is released within the lysosome of the target cell (Tang et al., 2019). Linkers are generally categorized as either cleavable or noncleavable. Noncleavable linkers primarily include thioethers and maleimidocaproyl moieties (Sheyi et al., 2022). Cleavable linkers can be further divided into chemically cleavable (e.g., hydrazones and disulfides) and enzymatically cleavable linkers (e.g., glucuronides and peptides) (Bargh et al., 2019). Within the cleavable category, the acid sensitivity (pH-responsive), enzyme sensitivity, or glutathione sensitivity of linkers may be exploited, depending on their structural features (Tang et al., 2019; Su et al., 2021). Cleavable linkers are intended to respond to the unique conditions of the tumour microenvironment, thereby facilitating controlled release of the payload. In contrast, noncleavable linkers remain inert under common chemical or enzymatic conditions, conferring improved plasma stability and minimizing off-target toxicity (Kovtun et al., 2006; Oflazoglu et al., 2008).

The cytotoxic payload represents the active component of an ADC, exerting its cytotoxic effects after internalization, and is typically a small-molecule drug (Jin et al., 2022). Given the limited bioavailability of ADCs—only ∼2% of the administered dose intravenously reaches the target tumour site (Beck et al., 2017)—the payload must be highly potent, with IC_50_ values usually in the nanomolar to picomolar range (Zhao et al., 2020). The efficacy of payload delivery depends on three principal factors: the abundance of accessible cell-surface antigens for antibody binding, the efficiency of antigen–antibody internalization, and the subsequent intracellular release of the cytotoxic drug. Currently, the most widely used payload classes include potent microtubule inhibitors, topoisomerase inhibitors, DNA-damaging agents, and immunomodulators (Diamantis and Banerji, 2016). An ideal ADC payload should combine high potency with excellent plasma stability, low immunogenicity, a small molecular weight, and a prolonged half-life (Li F. et al., 2016).

### 2.2 The value of innovative drug delivery strategies

#### 2.2.1 Precision targeting to reduce off-target toxicity

One of the core values of ADCs lies in chemically linking highly specific mAbs to potent cytotoxic drugs through linkers. This design enables the precise delivery of cytotoxic agents to tumour cells by leveraging the specificity of the antibody for target antigens while minimizing off-target effects on healthy tissues. ADCs can be used to overcome the low target specificity of traditional chemotherapies, thereby increasing therapeutic efficacy while minimizing side effects. In the DESTINY-CRC01 trial, CRC patients with HER2 expression (IHC 3+ or IHC 2+/ISH+) achieved a significant objective response rate (ORR) with T-DXd, whereas no objective responses were observed in patients with low HER2 expression levels (IHC 2+/IHC 1+/ISH−) (Yoshino et al., 2023). These results indicate that in addition to precise targeting by mAbs, ADCs can also align with target expression levels to support individualized treatment strategies.

#### 2.2.2 Smart linkers for personalized controlled release

Linker design enables responsive release triggered by tumour microenvironment (TME) conditions, such as acidic pH, enzyme activity, or reductive stress, which facilitates individualized control of release kinetics to adapt to tumour heterogeneity. Beyond classical cleavable linkers, researchers have developed smart responsive linkers that activate payload release by sensing specific TME features. For example, hypoxia-activated azobenzene linkers remain stable in normal tissues (O_2_ > 10%) but are cleaved in hypoxic tumour microenvironments (O_2_ < 1%), releasing monomethyl auristatin E (MMAE) and fully restoring ADC cytotoxicity (Xiao et al., 2024). In addition, differentiated release strategies using linker combinations have enabled temporally controlled release in dual-payload ADCs. For instance, KH815 employs a combination of irinotecan (a topoisomerase I inhibitor) and triptolide (an RNA polymerase II inhibitor). The former is rapidly released inside tumour cells via lysosomal enzyme-cleavable linkers, directly disrupting DNA replication, whereas the latter is triggered by pH-sensitive linkers in the TME to target stromal or vascular cells, achieving a “dual-hit” mode of tumour cell killing plus microenvironment remodelling (Mullard, 2025). Such innovations enable the release patterns of ADCs to be adjusted on the basis of individual tumour TME characteristics, laying the foundation for personalized therapy.

#### 2.2.3 Payload diversification to complement biomarker-driven stratification in CRC

Traditional ADCs have primarily relied on microtubule inhibitors, topoisomerase inhibitors, or DNA-damaging agents. In CRC, patient stratification is driven mainly by biomarker-defined targets (e.g., HER2, GCC, CEACAM5) and tumour biology, whereas payload class modulates efficacy and safety within these target-positive populations—particularly in the setting of heterogeneous antigen expression and acquired resistance. Payload diversification has therefore become a core strategy for improving the therapeutic index and overcoming heterogeneity/resistance (Beck et al., 2017; Conilh et al., 2023). Table 2 summarizes representative ADC payload classes and clinical selection considerations in CRC. In addition to conventional payloads, non-traditional payloads—including immunostimulatory agents (e.g., TLR/STING agonists), RNA polymerase inhibitors (e.g., amatoxins), and apoptosis modulators (e.g., Bcl-xL inhibitors)—may help address resistance by engaging antitumour immunity or targeting tolerant/dormant tumour-cell states (Wang et al., 2025b; Grairi and Le Borgne, 2024). Dual-payload ADCs (e.g., KH815) that combine mechanistically distinct warheads are also being explored to deliver a “dual-hit” effect on core tumour vulnerabilities such as replication and transcription (Mullard, 2025). Overall, payload diversification complements target-based stratification and is driving ADCs towards greater precision and efficacy in CRC.

**TABLE 2:** Representative payloads and supporting evidence for personalized therapy in CRC.

Payload class	Representative ADC	Mechanism	Evidence in CRC (clinical/preclinical)	Patient-selection considerations (target-driven)	Major toxicity and limitations	Refs
Topoisomerase I inhibitors (DXd, SN-38)	T-DXd; IMMU-130	DNA single-strand breaks; bystander effect	DESTINY-CRC01: ORR 45.3% in HER2-high mCRC; IMMU-130 showed activity in pretreated mCRC	Within biomarker-selected CRC (e.g., HER2+, CEACAM5+), membrane-permeable payloads with bystander effect can help heterogeneous antigen expression; for SN-38, consider UGT1A1 genotype when assessing diarrhoea/neutropenia risk	Diarrhoea, myelosuppression, off-tumour bystander effect	Dong et al. (2019), Siena et al. (2021)
Microtubule inhibitors (MMAE)	RC48 (disitamab vedotin)	Tubulin polymerization inhibition; cleavable linkers enable bystander killing	Preclinical CRC models are active; early clinical trials in HER2+ GI tumours	Best suited to biomarker-selected, moderate-to-high antigen expression; cleavable linker/bystander effect may partially offset heterogeneity; assess baseline neuropathy risk	Peripheral neuropathy, neutropenia	Wu et al. (2023a), Sheng et al. (2024)
DNA alkylators (Duocarmycin, Exatecan)	TAK-164; Precem-TcT	DNA alkylation/prodrug activation	TAK-164 active but hepatotoxicity; Precem-TcT Phase I: DCR 59%, mPFS 6.7 months in irinotecan-refractory CRC	Typically paired with high-expression targets (e.g., GCC, CEACAM5); tumour-activated linkers aim to improve selectivity; close liver/haematologic monitoring	Hepatotoxicity, haematologic toxicity	Abu-Yousif et al. (2020), Raab-Westphal et al. (2024)

#### 2.2.4 DAR regulation and pharmacokinetic optimization

The pharmacokinetic properties and overall therapeutic index of an ADC are profoundly influenced by its drug-to-antibody ratio (DAR) (Dumontet et al., 2023). The DAR defines the average number of payload molecules conjugated per antibody, and achieving an optimal balance is essential to ensure sufficient payload delivery without compromising antibody integrity (Chau et al., 2019). Early ADCs developed using conventional nonsite-specific conjugation methods typically yielded heterogeneous mixtures. Fractions with high DARs (≥4) exhibited stronger *in vitro* cytotoxic activity but exhibited increased hydrophobicity, resulting in accelerated clearance, shortened systemic exposure, and a narrower therapeutic window. In contrast, fractions with low DARs carried fewer payloads, leading to inadequate antitumour activity (Fu et al., 2022; Chis et al., 2024). The advent of site-specific conjugation strategies—such as engineered cysteine conjugation, enzymatic peptide ligation, and glycan-based conjugation—has enabled the generation of ADCs with homogeneous and controlled DARs, which are typically set at 2, 4, or 8 (Khongorzul et al., 2020; Samantasinghar et al., 2023; Choi et al., 2024; Li M. et al., 2024). These approaches allow precise and stoichiometric attachment at defined positions, markedly improving the homogeneity, stability, and activity of ADCs. Modern ADCs further integrate site-specific conjugation with tumour microenvironment–responsive linkers to achieve optimized pharmacokinetic profiles, maintaining stability and tolerability in systemic circulation while ensuring efficient payload release in tumour tissues, thereby broadening the therapeutic window (Drago et al., 2021).

## 3 ADC targets and therapeutic landscape in CRC

### 3.1 Clinically validated targets

#### 3.1.1 Target: HER2

HER2, also known as ErbB2, is a member of the EGFR family (Marchiò et al., 2021); HER2 forms heterodimers with HER3 to regulate specific signalling pathways within the tumour microenvironment, including activation of the PI3K/AKT/mTOR pathway (Díaz-Serrano et al., 2018; Miricescu et al., 2020), stimulation of the MAPK/ERK cascade through RAS activation (Su, 2018), and inhibition of GSK3β to maintain Wnt/β-catenin signalling (Liu et al., 2018; Kim Y. et al., 2023). These signalling cascades collectively govern tumour proliferation, epithelial–mesenchymal transition, metabolic reprogramming, therapeutic resistance, and metastasis (Shimozaki et al., 2024). HER2 also contributes to immune modulation. It induces the expression of cytokines such as interleukin-6, which activates the JAK/STAT pathway, leading to the upregulation of programmed death-ligand 1 (PD-L1) expression and promoting cancer stem cell self-renewal (Zhang et al., 2016; Hassan and Seno, 2022). Concurrently, HER2 signalling results in the recruitment of immunosuppressive cells, including regulatory T cells (Tregs) and M2-polarized macrophages, thereby reshaping the immune microenvironment to facilitate tumour immune evasion (Jing et al., 2023; Jiang et al., 2024). HER2 is overexpressed in multiple solid tumours, including gastric cancer (Joshi and Badgwell, 2021), colorectal cancer (Suwaidan et al., 2022), and breast cancer (Oh and Bang, 2020). Importantly, HER2 has emerged as one of the most successful therapeutic targets across diverse malignancies (Arteaga et al., 2011; La Salvia et al., 2019; Xie et al., 2020).

##### 3.1.1.1 RC48 (disitamab vedotin)

RC48 is an anti-HER2 ADC composed of a humanized anti-HER2 monoclonal antibody (hertuzumab) conjugated to the microtubule inhibitor MMAE via a cleavable linker (Yao et al., 2015; Li H. et al., 2016). It has been approved for the treatment of advanced or metastatic HER2-positive gastric cancer and urothelial carcinoma (Peng et al., 2021; Sheng et al., 2021; Hong X. et al., 2023; Zhou L. et al., 2025). Upon binding to HER2 on the cell membrane, RC48 undergoes internalization and lysosomal degradation, which may reduce the opportunity for STING to interact with HER2 and thereby facilitate activation of the cGAS–STING pathway (Wu et al., 2019; Wu X. et al., 2023). The antitumour activity of RC48 in CRC cells appears to be regulated by dual mechanisms: activation of cGAS–STING signalling enhances IFN-β secretion, promotes immune cell infiltration, and increases the cytotoxicity of tumour-infiltrating lymphocytes, whereas MMAE disrupts microtubules, leading to cell cycle arrest and apoptosis. Unlike conventional monoclonal antibodies, whose efficacy is strongly dependent on antigen expression levels, RC48 exhibits antitumour activity in CRC that appears less reliant on HER2 expression (Liu et al., 2024). This was supported clinically, as overall response rates were comparable between patients with low and high HER2 expression, and was consistent with preclinical evidence showing that RC48 activity in CRC PDX models is not correlated with HER2 expression (Wu X. et al., 2023; Sheng et al., 2024). Further mechanistic studies revealed that in the HT29 and SW480 cell lines, RC48 may induce senescence in both HER2-high and HER2-low CRC cells by upregulating CDKN1A protein expression (Wu et al., 2025). Multiple ongoing prospective clinical trials are currently evaluating the efficacy and safety of RC48 as a second-line treatment for HER2-positive advanced CRC (NCT05785325, NCT05493683, and NCT05578287).

##### 3.1.1.2 T-DM1 (trastuzumab emtansine)

T-DM1 is generated by conjugating the high-affinity humanized anti-HER2 monoclonal antibody trastuzumab with the small-molecule microtubule inhibitor mertansine (DM1) through a maleimide methyl cyclohexanecarboxylate linker (Tarantino and Tolaney, 2022). T-DM1 retains the ADCC of trastuzumab, while its conjugated DM1 is released upon internalization and lysosomal degradation, blocking microtubule polymerization and inducing mitotic catastrophe (Barok et al., 2014). *In vitro* experiments confirmed that compared with cetuximab or trastuzumab, T-DM1 inhibited LS174T and HT-29 CRC cells more effectively. In a xenograft mouse model, T-DM1 combined with metformin significantly suppressed tumour growth (Chung et al., 2020). The multicentre phase II HERACLES-B trial further revealed that pertuzumab plus T-DM1 exhibited antitumour activity in HER2-positive metastatic CRC (mCRC), particularly in patients with high HER2 immunohistochemistry (IHC) scores (Sartore-Bianchi et al., 2020).

##### 3.1.1.3 T-DXd (DS-8201a, trastuzumab deruxtecan)

T-DXd, also known as DS-8201 or trastuzumab deruxtecan, is an ADC that links the topoisomerase I inhibitor DXd to trastuzumab via a tetrapeptide-based cleavable linker. Its therapeutic efficacy in HER2-expressing or HER2-mutant tumours is attributed to several key features: the highly active topoisomerase I inhibitor payload, a stable and specific tetrapeptide-cleavable linker, a high DAR of up to 8, and a strong bystander effect (Ogitani et al., 2016; Takegawa et al., 2019). Currently, T-DXd has been approved in multiple countries for the treatment of metastatic HER2-positive breast cancer and gastric cancer (Keam, 2020).

T-DXd has demonstrated substantial clinical activity with a manageable safety profile in HER2-positive metastatic CRC, establishing it as a leading HER2-directed ADC. The DESTINY-CRC01 study, a multicentre phase II clinical trial, reported the efficacy and safety of T-DXd in patients with HER2-positive mCRC (Siena et al., 2021). The results demonstrated an ORR of 45.3%, which is particularly notable in HER2-positive mCRC patients who had progressed after at least two prior lines of therapy. The most common grade ≥3 treatment-emergent adverse events were neutropenia and anaemia, which were generally manageable (Tsurutani et al., 2020; Yoshino et al., 2023). Subsequent DESTINY-CRC02 findings further confirmed that 5.4 mg/kg is the optimal monotherapy dose of T-DXd for treating HER2-positive mCRC (Raghav et al., 2024).

##### 3.1.1.4 SHR-A1811

SHR-A1811 is an ADC composed of trastuzumab, a cleavable linker, and a novel topoisomerase I inhibitor, SHR169265 (Zhang et al., 2025). SHR-A1811 showed promising antitumour activity in heavily pretreated, unresectable, or metastatic solid tumours (Li Z. et al., 2024; Li J. J. et al., 2025), with a low incidence and severity of interstitial lung disease. With an optimized DAR value of 6, SHR-A1811 retains strong cytotoxic activity and a favourable bystander killing effect, providing antitumour efficacy comparable to that of T-DXd while improving plasma stability *in vitro*. In preclinical models, SHR-A1811 demonstrated dose-dependent antitumour activity and favourable safety. The safety and efficacy of SHR-A1811 in patients with HER2-expressing or HER2-mutant advanced solid tumours were evaluated in a multicentre, dose-escalation phase I clinical trial (NCT04446260) (Yao et al., 2024). Among the 11 enrolled CRC patients, 4 (36.4%) achieved objective responses. Multiple ongoing clinical trials are being conducted to further assess the efficacy and safety of SHR-A1811 in advanced CRC patients refractory to oxaliplatin, 5-fluorouracil, and irinotecan (NCT06199973, NCT04513223, and NCT06666166).

##### 3.1.1.5 Limitations and future directions

HER2-targeted ADCs are among the most clinically advanced ADC strategies in CRC, but the evidence base is still largely derived from single-arm phase II cohorts in highly selected HER2-positive populations. In DESTINY-CRC01, trastuzumab deruxtecan produced objective responses primarily in IHC 3+/ISH+ disease, whereas activity was markedly lower in HER2-low cohorts, highlighting the impact of assay definition and intratumoural heterogeneity on patient selection (Siena et al., 2021). DESTINY-CRC02 confirmed activity at 5.4 mg/kg with improved tolerability versus 6.4 mg/kg, yet interstitial lung disease/pneumonitis remained a clinically meaningful risk that requires proactive monitoring and standardized management (Powell et al., 2022; Raghav et al., 2024). Finally, prior experience with anti-HER2 therapy in mCRC suggests that co-alterations and heterogeneous HER2 amplification may contribute to primary or acquired resistance, supporting composite biomarker strategies and rational combinations rather than direct extrapolation from breast/gastric paradigms (Sartore-Bianchi et al., 2020). A consolidated comparison is provided in Table 3.

**TABLE 3:** Integrated overview of ADCs by target in CRC.

Target	ADC	Payload/linker/DAR	Clinical phase	CRC setting/population	Key efficacy outcomes	Key safety/limitations/status	Key references
HER2	RC48 (Disitamab Vedotin)	MMAE, cleavable linker; DAR ∼4	Phase II (single-arm)	Later-line HER2-expressing or HER2-amplified mCRC (typically third-line or later)	Small single-arm cohort reported: ORR 13.6%, DCR 77.3%, mPFS 4.11 months, mOS 10.45 months	Evidence based on a small sample size; MMAE-related peripheral neuropathy and myelosuppression; TKI-related hypertension and hand-foot syndrome	Sheng et al. (2024), Vaghi et al. (2025), Wu et al. (2025)
HER2	T-DM1(Trastuzumab Emtansine)	DM1, largely non-cleavable linker; DAR ∼3.5	Phase II (single-arm)	Heavily pretreated HER2-amplified/overexpressing mCRC	Reported low confirmed ORR (∼9–10%) with disease stabilization in a subset; mPFS around 4 months in published phase II experience	Modest objective response in CRC; typical toxicities include thrombocytopenia and transaminase elevation; not a preferred option when higher-activity anti-HER2 strategies are available	Sartore-Bianchi et al. (2020)
HER2	T-DXd(DS-8201a, Trastuzumab deruxtecan)	DXd, cleavable linker; DAR ∼8	Phase II (single-arm)	Previously treated metastatic HER2-positive CRC; benefit mainly in HER2 IHC 3+ (limited activity in lower expression cohorts)	DESTINY-CRC01 Cohort A: ORR 45.3%, DCR 83.0%, mPFS 6.9 months, mOS 15.5 months; DESTINY-CRC02: ORR 37.8% (5.4 mg/kg) and 27.5% (6.4 mg/kg), with 5.4 mg/kg generally showing a more favorable risk-benefit profile	ILD/pneumonitis requires active monitoring and early intervention; common toxicities include gastrointestinal events and myelosuppression; efficacy is strongly associated with high HER2 expression	Siena et al. (2021), Raghav et al. (2024)
HER2	SHR-A1811	Top1i payload, cleavable linker; DAR ∼6	Phase I (dose-escalation)	Advanced HER2-positive CRC included in expansion cohorts after failure of standard therapies	Antitumor activity in CRC has been described in meeting presentations	CRC evidence remains early; safety profile needs to mature	Li et al. (2024d), Yao et al. (2024), Li et al. (2025a), Vaghi et al. (2025)
HER3	U3-1402 (Patritumab deruxtecan)	DXd, cleavable linker; DAR ∼8	Phase II (single-arm)	Previously treated advanced/metastatic HER3-expressing CRC (tumor-agnostic program with CRC enrollment)	Preclinical (CRC): Marked tumour regression in HER3-high CRC xenografts (DiFi, SW620), but no effect in HER3-low Colo320DM, indicating HER3 expression–dependent activity. Clinical (CRC): NR	CRC-specific peer-reviewed efficacy readouts are not yet publicly available; CRC results from the ongoing phase II study are pending, and further CRC-focused validation will be required once reported	Koganemaru et al. (2019)
HER3	DB-1310	Top1i, cleavable linker; DAR ∼8	Phase I/IIa (dose-escalation/expansion)	Advanced solid tumors	Preclinical (CRC-relevant): Potent cytotoxicity in multiple HER3+ tumour models and *in vivo* antitumour activity, including colon xenograft efficacy in preclinical testing	Early dose-escalation stage; CRC-stratified efficacy is not yet established in publicly available datasets	Li et al. (2024c); Li et al. (2025b)
HER3	AMT-562	Exatecan, self-immolative linker; DAR NR	Phase I (dose-escalation)	Advanced solid tumors	Preclinical (CRC): Demonstrated deep and durable antitumour responses across multiple CRC CDX/PDX models, including models insensitive to patritumab-DXd comparators in the same study. Clinical (CRC): NR	Registration/early clinical information available; human CRC efficacy outcomes have not been sufficiently reported for firm comparisons	Weng et al. (2023a)
GCC	TAK-164	IGN, peptide linker; DAR ∼2.6	Phase I (dose-escalation)	Heavily pretreated GCC-positive mCRC	Reported very low ORR (single unconfirmed PR) with DCR driven largely by stable disease in early-phase experience	Development was limited by safety concerns, including serious hepatotoxicity; overall risk-benefit did not support continued development in CRC.	Abu-Yousif et al. (2020), Kim et al. (2023a)
GCC	TAK-264 (MLN0264)	MMAE, protease-cleavable linker; DAR ∼4.3	Phase I (dose-escalation)	GCC-expressing advanced gastrointestinal malignancies (including CRC)	Overall objective responses were rare; reported activity was limited, with most patients achieving stable disease at best in early clinical evaluation	Limited antitumor activity in CRC; MMAE-class toxicities (e.g., myelosuppression, neuropathy) and gastrointestinal adverse events were observed	Almhanna et al. (2016)
CEACAM5	IMMU-130 (Labetuzumab govitecan)	SN-38, proprietary linker; DAR ∼7.6	Phase I/II (dose-escalation/expansion)	Refractory mCRC (typically irinotecan-pretreated)	Low confirmed ORR (rare PR) with disease stabilization in a subset; reported mPFS ∼3.6 months and mOS ∼6.9 months in published experience	Main toxicities included myelosuppression and diarrhea, consistent with SN-38 exposure; limited objective response as a late-line monotherapy	Dotan et al. (2017)
CEACAM5	Precemtabart tocentecan (Precem-TcT, M9140)	Exatecan, beta-glucuronide linker; DAR 8	Phase I (dose-escalation)	Heavily pretreated mCRC, including irinotecan-resistant disease	Confirmed ORR 7.5% (3/40); overall mPFS 5.9 months; mPFS 6.7 months in patients receiving >=2.4 mg/kg; MTD 2.8 mg/kg q3w	DLTs were predominantly hematologic; no ILD or ocular toxicity was reported in the published phase I dataset	Kopetz et al. (2025b)
CEACAM5	SAR408701 (Tusamitamab ravtansine)	DM4, cleavable linker; DAR ∼3.8	Phase I (dose-escalation)	CEACAM5-expressing solid tumors	Antitumor activity varies across tumor types; CRC-specific efficacy has not been firmly established in publicly available data	DM4-ADC class is often associated with ocular toxicity (e.g., keratopathy); CRC development status remains exploratory	Gazzah et al. (2022)

#### 3.1.2 Target: HER3

HER3, also known as ErbB3, is another member of the EGFR family (Sierke et al., 1997) and is overexpressed in various solid tumours, including CRC (Campbell et al., 2010). HER3 can be phosphorylated through heterodimerization with other receptor tyrosine kinases, such as HER2(Olayioye et al., 2000; Campbell et al., 2010), thereby activating the PI3K/AKT and MAPK/ERK signalling pathways (Yarden and Sliwkowski, 2001; Baselga and Swain, 2009; Ocana et al., 2012; Rathore et al., 2025). Given its high expression in tumours and efficient internalization upon antibody binding, HER3 is considered a promising target for ADC development(Haikala and Jänne, 2021).

##### 3.1.2.1 U3-1402 (patritumab deruxtecan)

U3-1402 is a HER3-targeted ADC composed of the fully humanized anti-HER3 monoclonal IgG1 antibody patritumab conjugated to DXd via a tetrapeptide-based linker (Hashimoto et al., 2019). The antitumour activity of U3-1402 is correlated with HER3 expression levels but is independent of Kirsten rat sarcoma viral oncogene homolog (KRAS) mutation status (Koganemaru et al., 2019). After internalization, U3-1402 releases DXd, which enters the nucleus and induces DNA damage, thereby exerting cytotoxic effects (Yonesaka et al., 2019). In xenograft mouse models, U3-1402 demonstrated both dose-dependent and HER3-dependent antitumour activity. Notably, U3-1402 enhanced the infiltration of innate and adaptive immune cells, increasing the sensitivity of HER3-expressing tumours to programmed death receptor 1 (PD-1) blockade (Haratani et al., 2020). Toxicological studies have also confirmed the acceptable safety profile of U3-1402. A phase II trial evaluating U3-1402 in patients with advanced or mCRC (NCT04479436) has now advanced to the second stage.

##### 3.1.2.2 DB-1310

DB-1310 is an anti-HER3 ADC in which the humanized anti-HER3 antibody Hu3f8 is covalently conjugated to a proprietary DNA topoisomerase I inhibitor, P1021, through a cleavable linker (Li X. et al., 2024). Overall, DB-1310 demonstrated dose-dependent antitumour activity and good biosafety in preclinical studies. This unique linker–payload system increases the hydrophilicity of the ADC, thereby allowing a higher DAR of up to 8. *In vitro* studies have shown that DB-1310 exhibits high binding affinity for HER3, efficient internalization, and a favourable bystander effect (Li X. et al., 2025). A first-in-human phase I/IIa dose-escalation and expansion trial is currently ongoing to evaluate the safety and tolerability of DB-1310 in patients with advanced solid tumours (NCT05785741).

##### 3.1.2.3 AMT-562

AMT-562 is generated by conjugating a novel anti-HER3 antibody (Ab562) with exatecan (Weng et al., 2023a). Preclinically, in CRC PDX models, compared with other ADCs, AMT-562 demonstrated superior tumour suppression, particularly in rectal cancer models with relatively high HER3 expression. Exatecan, a precursor of DXd, shows greater potency, greater cell permeability, less sensitivity to multidrug resistance, and a stronger bystander killing effect than DXd does (Joto et al., 1997; Weng et al., 2023b). In addition, the synergy of AMT-562 with other treatment regimens may become a new strategy for overcoming multidrug resistance. A first-in-human phase I dose-escalation trial is currently underway to evaluate the safety and tolerability of AMT-562 in patients with advanced solid tumours (NCT06199908).

##### 3.1.2.4 Limitations and future directions

Although HER3 is broadly expressed and may increase in advanced/metastatic CRC, expression alone does not guarantee clinical tractability for an ADC. In patient cohorts, membranous HER3 expression has been reported to be substantially higher in metastatic CRC than in early-stage disease and associated with adverse outcomes in some subgroups (Capone et al., 2023), providing a biologic rationale for targeting. However, the most mature clinical experience with patritumab deruxtecan (HER3-DXd) comes from non-CRC settings; for example, a multicenter phase I/II trial in metastatic breast cancer showed durable responses across HER3-high and HER3-low tumors but also a non-negligible risk of interstitial lung disease, including fatal events (Krop et al., 2023). Together with earlier phase I data demonstrating dose-limiting hematologic/hepatic toxicities for HER3-DXd (Tsurutani et al., 2020), these findings suggest that translation to CRC will likely depend on rigorous biomarker-driven enrichment (e.g., quantitative antigen density, internalization signatures) and careful safety mitigation rather than assuming that ubiquitous HER3 expression is sufficient. A consolidated comparison is provided in Table 3.

### 3.2 Emerging and exploratory targets

#### 3.2.1 Target: EGFR

Human epidermal growth factor receptor (EGFR), also known as ErbB1/HER1, is a member of the ErbB family (Kumagai et al., 2021). In the absence of specific ligands, EGFR remains inactive (Yarden and Sliwkowski, 2001). The binding of ligands to the extracellular domain induces homodimerization or heterodimerization with other ErbB RTKs, triggering phosphorylation of the tyrosine kinase domain. This activates downstream signalling pathways, including the RAS/RAF/MAPK pathway (Hynes and Lane, 2005) and the PI3K/AKT pathway (Glaviano et al., 2023). Currently, anti-EGFR monoclonal antibodies such as cetuximab and panitumumab are effective only in a small subset of cancer patients (Ciardiello and Tortora, 2008; Bokemeyer et al., 2011; Douillard et al., 2014; Van Cutsem et al., 2015). Therefore, EGFR-targeted ADCs may represent a novel strategy to overcome these limitations.

##### 3.2.1.1 ABBV-321 (serclutamab talirine)

ABBV-321 is an EGFR-targeting ADC that couples the affinity-matured antibody AM1 with a pyrrolobenzodiazepine (PBD) dimer (Anderson et al., 2020; Carneiro et al., 2023). ABBV-321 may extend therapeutic activity to colorectal tumours with low-to-moderate EGFR expression. PBD dimer is a DNA cross-linker with significantly stronger antitumour activity and a more potent bystander effect compared with those of clinically validated payloads such as MMAE or maytansinoids. Since colorectal tumours often express relatively low levels of EGFR, most cell lines are largely insensitive to MMAE-based ADCs. However, ABBV-321 has demonstrated notable cytotoxic activity in colorectal cancer cell lines and in xenograft models (Anderson et al., 2020). A phase I study evaluating the safety, pharmacokinetics, and antitumour activity of ABBV-321 in patients with advanced solid tumours associated with EGFR overexpression was completed (NCT03234712).

##### 3.2.1.2 Limitations and future directions

EGFR is a validated target for naked antibodies in RAS wild-type CRC, but ADC approaches have not yet demonstrated clear benefit in CRC, underscoring that target “validation” does not automatically translate to ADC success. In the first-in-human trial of the EGFR-ADC MRG003, the colorectal cancer expansion cohort reported an objective response rate of 0% (disease control rate 25%), implying that EGFR IHC positivity alone may be an insufficient enrichment strategy (Qiu et al., 2022). In parallel, on-target/off-tumour toxicity remains a central limitation for EGFR ADCs: depatuxizumab mafodotin (ABT-414) was associated with eye-related adverse events in 91% of EGFR-amplified glioblastoma patients and grade 3/4 ocular events in 33% (van den Bent et al., 2017). For CRC, these data argue for a narrower therapeutic window and motivate next-generation design choices—payloads/linkers with improved tolerability, prophylactic eye-care protocols, and biomarker strategies beyond EGFR expression (e.g., amplification/internalization or conditional activation) to justify continued development.

#### 3.2.2 Target: GCC

Guanylyl cyclase C (GCC) is the receptor for diarrhoea-inducing heat-stable enterotoxins as well as the endogenous ligands guanosine and uridine (Waldman and Camilleri, 2018). In normal gastrointestinal tissue, GCC expression is restricted to the apical surface of epithelial cells at tight junctions, where it is isolated from systemic circulation (Carrithers et al., 1996). Upon ligand binding, GCC undergoes receptor-mediated endocytosis (Urbanski et al., 1995). In contrast, GCC is expressed in more than 95% of primary and metastatic colorectal cancers and in approximately 65% of oesophageal, gastric, and pancreatic tumours (Urbanski et al., 1995; Waldman et al., 1998; Buc et al., 2005). As such, the GCC has attracted interest as a potential ADC target.

##### 3.2.2.1 TAK-164

TAK-164 is a GCC-targeted ADC in which the highly cytotoxic DNA alkylator DGN549 is conjugated to a human anti-GCC monoclonal antibody via a peptide linker (Abu-Yousif et al., 2020). Preclinical studies have confirmed that TAK-164 selectively binds, internalizes, and induces potent cytotoxicity in GCC-expressing cells. A single intravenous dose of TAK-164 (0.76 mg/kg) significantly inhibited tumour growth in an mCRC primary human tumour xenograft model. Imaging studies further demonstrated a positive correlation between GCC expression and TAK-164 tumour uptake. However, the results from a multicentre phase I dose-escalation trial indicated dose-limiting hepatotoxicity at higher levels and insufficient clinical benefit, leading to the discontinuation of the study (Kim R. et al., 2023).

##### 3.2.2.2 TAK-264 (MLN0264)

TAK-264, also known as MLN0264, is a GCC-targeted ADC in which a fully human anti-GCC monoclonal antibody is conjugated to MMAE via the protease-cleavable peptide maleimido-caproyl-valine-citrulline (VC) (Gallery et al., 2018). *In vitro* studies confirmed its selective binding, internalization, and cytotoxicity against GCC-expressing cells. *In vivo*, TAK-264 demonstrated dose-dependent antitumour activity and favourable tolerability in HEK293-GCC-engineered models and human primary tumour xenografts. In a first-in-human phase I trial, 35 patients were enrolled, the majority (85%) of whom had mCRC. While TAK-264 was generally well tolerated and safe, no clear antitumour activity or clinical benefit was observed in mCRC (Almhanna et al., 2016).

##### 3.2.2.3 Limitations and future directions

GCC expression is highly enriched in intestinal epithelium and is retained in many CRCs, making it an attractive antigen; however, clinical experience with GCC-directed ADCs highlights the complexity of apparently “restricted” targets. In a phase I study of TAK-264 (MLN0264), antitumor activity was limited despite GCC expression, and gastrointestinal toxicities such as diarrhea were common, indicating that normal gut expression can still translate into a constrained therapeutic index (Almhanna et al., 2016). More recently, TAK-164 also showed limited efficacy together with notable GI adverse events, reinforcing that GCC targeting may require alternative payload/linker choices or locally activated approaches to improve the balance between efficacy and safety (Kim R. et al., 2023). A consolidated comparison is provided in Table 3.

#### 3.2.3 Target: CEACAM5

Carcinoembryonic antigen-related cell adhesion molecule-5 (CEACAM5), also known as CEA or CD66e, is a well-established diagnostic marker in CRC (MacSween et al., 1972; Beauchemin and Arabzadeh, 2013). CEACAM5 is also highly expressed in multiple malignancies, such as pancreatic cancer, gallbladder cancer, non-small cell lung cancer, and bladder cancer, but its expression in normal tissues is limited (Eades-Perner et al., 1994; Beauchemin and Arabzadeh, 2013). As a serum biomarker, CEACAM5 demonstrated the highest sensitivity (93.7%) and specificity (96.1%) in a CRC cohort (Tiernan et al., 2013). Its tumour-specific overexpression makes CEACAM5 an ideal target for ADC-based therapies.

##### 3.2.3.1 IMMU-130 (labetuzumab govitecan)

IMMU-130 (labetuzumab govitecan) is an ADC that targets CEACAM5. Clinically, in patients with advanced, refractory, or relapsed mCRC, IMMU-130 demonstrated promising therapeutic activity. It couples the humanized anti-CEACAM5 monoclonal antibody labetuzumab with the moderately cytotoxic topoisomerase I inhibitor SN-38 via a relatively stable CL2A linker, with a high DAR of 7–8 (Moon et al., 2008; Govindan et al., 2009; Dong et al., 2019). IMMU-130 significantly inhibited tumour growth in GW-39 human colon cancer lung metastasis models and subcutaneous LS174T xenograft models in nude mice (Govindan et al., 2009). Two independent phase I clinical trials have been initiated. Among 86 patients who had received five prior lines of therapy, 38% experienced tumour shrinkage and a decline in CEA levels; one patient achieved a partial response lasting more than 2 years, and 42 patients had stable disease, representing the best overall disease control (Dotan et al., 2017). Neutropenia was the only major controllable adverse event (Segal et al., 2014). A phase II clinical study of IMMU-130 in metastatic CRC patients is currently ongoing (Govindan et al., 2015).

##### 3.2.3.2 Precemtabart tocentecan

Precemtabart tocentecan (Precem-TcT, previously M9140) is the first clinical-stage TOP1 inhibitor–based ADC targeting CEACAM5 and was generated by conjugating a CEACAM5-specific antibody with exatecan (Raab-Westphal et al., 2024). Preclinical studies have shown potent antitumour activity and pronounced bystander effects. In the dose-escalation stage of the ongoing phase I trial (PROCEADE-CRC-01), 40 heavily pretreated, irinotecan-refractory mCRC patients received precemtabart tocentecan once every 3 weeks. The results revealed a disease control rate (DCR) of 58.8% (with a confirmed partial response rate of 8.8%) and a median progression-free survival (mPFS) of 6.7 months (95% CI: 4.6–8.8), indicating encouraging antitumour efficacy (Kopetz et al., 2025a). The main adverse events were haematologic toxicities, which were largely manageable with appropriate interventions. Notably, no interstitial lung disease or ocular toxicity was reported.

##### 3.2.3.3 SAR408701 (tusamitamab ravtansine)

SAR408701 is a novel ADC that targets CEACAM5, in which the anti-CEACAM5 antibody SAR408377 is covalently linked to the cytotoxic maytansinoid DM4. In CRC PDX models, SAR408701 showed robust antitumour activity with a clear dose–response relationship, and compared with a single dose, repeated administration resulted in greater responses (Decary et al., 2020). In the first-in-human dose-escalation study, 18 patients with CRC were treated with SAR408701 (Gazzah et al., 2022). Objective responses were observed in 3 patients (9.7%), including two patients with ≥2+ CEACAM5 expression on 100% of tumour cell membranes. In terms of safety, the incidence and severity of haematologic toxicity were lower than those associated with standard antimicrotubule agents such as docetaxel (Guastalla and Diéras, 2003). The dose-limiting toxicity of SAR408701 was reversible keratopathy.

##### 3.2.3.4 Limitations and future directions

CEACAM5 is highly prevalent in CRC, but heterogeneous expression and antigen shedding can create an “antigen sink” and reduce effective tumor exposure, complicating ADC translation. In a phase I study of the CEACAM5-directed ADC labetuzumab govitecan (SN-38), clinical activity in refractory mCRC was modest and key toxicities (notably neutropenia and diarrhea) reflected systemic payload exposure (Dotan et al., 2017). These results suggest that CEACAM5-ADC development may benefit from next-generation designs that better separate tumor versus normal-tissue exposure (e.g., optimized linker stability and bystander effect) and from biomarker strategies that go beyond IHC positivity to incorporate quantitative antigen density and shedding dynamics. A consolidated comparison is provided in Table 3.

#### 3.2.4 Emerging targets

In addition to HER2, HER3, EGFR, GCC, and CEACAM5, several emerging targets, including CDH17, PD-L1, and LGR5, have recently been investigated in CRC. These targets are characterized by relatively specific expression profiles and are often closely associated with tumour initiation and progression, immune evasion, stem cell maintenance, and drug resistance. Although the number of ADCs developed against these targets remains limited, most agents still at the preclinical or early clinical stage have already demonstrated certain antitumour potential. To provide a more straightforward overview of these advances, representative information on the corresponding ADCs is summarized in Table 4.

**TABLE 4:** Emerging targets and representative ADCs in CRC.

Target	ADC	mAbs	Payload	Preclinical/Clinical stage	Key findings	Ref.
CDH17	7MW4911	Mab0727	MF-6	Preclinical, xenograft	Potent anti-tumour activity in CRC CDX/PDX models, including multidrug-resistant settings; demonstrated bystander killing in a CDH17+/CDH17− co-culture assay	Wang et al. (2025a)
PD-L1	anti-PD-L1-PBA-Cur	anti-PD-L1	curcumin	Preclinical, xenograft	The uptake of curcumin was improved while the stability of PD-L1 was reduced, and immune escape was inhibited	Ding et al. (2025)
	aPDL1-NPLG-SN38	aPDL1	SN-38	Preclinical, xenograft	DAR up to 72	Zhang et al. (2023)
LGR5	anti-LGR5–vc-MMAE,anti-LGR5–NMS818	hu8E11v2	MMAE and NMS818	Preclinical, GEMM and xenograft	It had reasonable specificity and did not affect the steady-state intestinal epithelium or other tissues expressing LGR5	Junttila et al. (2015)
	Anti-LGR5-mp-MMAE,anti-LGR5 mAb-mcvc-PAB-MMAE	anti-LGR5 mAb	MMAE	Preclinical, xenograft	LGR5 expression-dependent antitumour effect, while reducing tumour recurrence	Gong et al. (2016)
CEACAM6	84-EBET	#84.7	EBET	Preclinical, xenograft	Participated in the regulation of CAFs, synergistic antitumour effects	Nakazawa et al. (2024), Kogai et al. (2025)
EREG	EREG ADC	H231	Duocarmycin DM	Preclinical, xenograft	The highly selective killing effect was unrelated to the MSI-H/MSS subtype	Jacob et al. (2025)
CLDN1	6F6-ADC	6F6	MMAE	Preclinical, xenograft	Oxaliplatin sequential therapy had a synergistic effect	Cherradi et al. (2017), Cherradi et al. (2023)
DR5	Oba01	zaptuzumab	MMAE	Preclinical, xenograft	Joint Abem had a synergistic effect	Zhang et al. (2021), Zheng et al. (2023), Zhou et al. (2025a)
CD47	7DC-DM1 ADC	7DC mAb	DM1	Preclinical, xenograft	Strong targeting and an immune-killing effect	Chiang et al. (2025)
Claudin-2	PNU-conjugated anti-Claudin-2 ADC	Anti-Claudin-2 Ab	PNU	Preclinical, xenograft	Suppressed liver metastasis; moderate immune activation without liver toxicity	Tabariès et al. (2024)
CD98hc	anti-CD98hc-DM1	anti-CD98hc^ECTO^ antibody	DM1	Preclinical, PDO and xenograft	Strong antiproliferative effect; mitotic arrest; good safety	Montero et al. (2023)
DOG1	anti-DOG1-DM-ADC	anti-DOG1 antibody	DM4	Preclinical, xenograft and liver metastasis	Inhibition of colon cancer growth and liver metastasis	Wu et al. (2023b)
GPR56	GPR56 ADC	10C7	DMSA	Preclinical, organoid and xenograft	Selective cytotoxicity in GPR56^+^ cells; strong efficacy	Jacob et al. (2023)
CDCP1	ch10D7-MMAE	ch10D7	MMAE	Preclinical, xenograft	Growth inhibition in CDCP1^+^ CRC, minimum threshold for efficacy	Khan et al. (2022)
ADAM9	IMGC936	MGA021	DM21-C	Phase I/II (NCT04622774)	Efficacy in CRC PDX; safe in primates	Scribner et al. (2022)
cMet	TR1801-ADC	anti-MET antibody	Tesirine	Phase I (NCT03859752)	Potent efficacy in CRC PDX; activity independent of MET CNV	Gymnopoulos et al. (2020), Cazes et al. (2021)
GOLPH2	G2-2-PBD	G2-2	Tesirine	Preclinical, PDX	High efficacy; expression was stable after therapy	Liewen et al. (2019)
5T4	H6-DM4	H6	DM4	DM4 Preclinical, xenograft	Efficacy correlated with 5T4 expression *in vivo*; eliminated CIC-induced tumours; active against platinum-resistant CRC	Wang et al. (2018)
Cripto	BIIB015	huB3F6	DM4	Phase I (NCT00674947)	Cripto-dependent efficacy; synergistic with chemotherapy	Kelly et al. (2011)
RON	Zt/g4–DM1	Zt/g4	DM1	Preclinical, xenograft	>90% tumour suppression; durable efficacy; good safety	Feng et al. (2014)

### 3.3 ADC combination strategies

Owing to the complexity of cancer treatment caused by tumour heterogeneity and drug resistance, multiple therapeutic approaches are often combined in clinical practice to improve the likelihood of remission and cure. The primary methods for increasing the efficacy of ADCs or overcoming resistance involve combining ADCs with other therapeutic strategies, such as chemotherapy, targeted agents, and immunotherapy.

#### 3.3.1 ADCs combined with chemotherapy

Rational combinations of antibody–drug conjugates (ADCs) with conventional chemotherapy are increasingly explored to deepen responses and delay or overcome resistance, provided that the partner drug adds complementary antitumor pressure without duplicating the ADC’s dose-limiting toxicities (Pretelli et al., 2024; Wei et al., 2024). Because most ADCs require binding, internalization, and intracellular processing to release active payload, the sequence of administration can meaningfully affect efficacy: preclinical data suggest that sequential dosing can produce greater tumor cell damage than concurrent administration (Wei et al., 2024). Despite these potential benefits, the major barrier to clinical development remains tolerability, as ADC–chemotherapy combinations frequently exacerbate grade ≥3 adverse events, consistent with overlapping systemic toxicities driven by off-target/on-target off-tumor exposure to payload (Tan et al., 2025). This risk is further shaped by ADC design features such as linker stability and DAR, which influence systemic free payload exposure and correlate with severe toxicities, underscoring the need for careful partner selection and schedule/dose optimization in combination regimens (Markides et al., 2024; Tang S. C. et al., 2024; Tan et al., 2025).

In both preclinical and clinical studies, combining different forms of chemotherapy with ADCs has been widely recognized as an effective strategy to overcome resistance and achieve favourable therapeutic outcomes (Yardley, 2013). The synergistic interactions between chemotherapy and ADCs include targeting different stages of the cell cycle or modulating the expression of tumour cell surface antigens. The sequence of administration plays a critical role in treatment efficacy, which may be related to the efficiency of ADC internalization (Wahl et al., 2001). However, the major challenge limiting the clinical development of these combinations is the significant increase in toxicity, which is likely caused by overlapping toxicity from the off-target and off-tumour effects of ADC payloads (Martin et al., 2016; Cortés et al., 2020).

In preclinical models, RC48 combined with gemcitabine demonstrated synergistic antitumour activity both *in vitro* and *in vivo*. Further RNA-seq analyses indicated that the combination may have synergistic effects on CRC cells through the regulation of multiple signalling pathways, such as the p53, PI3K-AKT, and MAPK pathways and pathways involved in the cell cycle(Liu et al., 2024). Another study revealed that Oba01 (an ADC targeting DR5) combined with the CDK inhibitor abemaciclib significantly enhanced the *in vivo* inhibition of microsatellite stability (MSS) and microsatellite instability-high (MSI-H) CRC PDX growth without inducing toxicity or resistance (Zhou D. et al., 2025). In rectal cancer PDX models, AMT-562 initially showed promising efficacy, but the tumours quickly relapsed; the combination of AMT-562 with rabusertib resulted in more durable antitumour effects (Weng et al., 2023a). Collectively, these findings highlight the potential of combining ADCs with conventional chemotherapeutics as novel treatment strategies for advanced CRC, particularly the refractory MSS subtype. For patients with a heavy tumour burden and rapid progression, ADC–chemotherapy combinations can achieve rapid tumour control.

#### 3.3.2 ADCs combined with targeted therapy

Targeted therapies—including monoclonal antibodies, tyrosine kinase inhibitors, and antiangiogenic agents—have proven clinical safety and efficacy in tumours with specific mutations, overexpression, or amplification. However, resistance and clonal heterogeneity narrow the therapeutic window of targeted monotherapies, leading to the emergence of ADC–targeted therapy combinations. The goal is to achieve more potent inhibition of oncogene-dependent signalling pathways, increase the availability of surface antigens, and sensitize tumours with low antigen expression. Synergistic mechanisms include improving intratumoural drug delivery via antiangiogenic effects (Ponte et al., 2016; Bordeau et al., 2021), modulating tumour antigen expression (La Monica et al., 2017; Haikala et al., 2022), overcoming intratumoural heterogeneity and resistance (Saatci et al., 2018), and inducing synthetic lethality.

Combining ADCs with targeted agents—such as anti-angiogenic therapies—may enhance antitumour activity in CRC and warrants clinical evaluation. As a monotherapy, AMT-562 showed limited efficacy in CRC, but when it was combined with bevacizumab or cetuximab, it demonstrated significantly increased antitumour activity (Weng et al., 2023a). Currently, clinical trials of RC48 in combination with bevacizumab or tislelizumab are underway in patients with HER2-positive advanced CRC, aiming to further evaluate the efficacy and safety of the combination treatment in a second-line setting (NCT05785325, NCT05493683). The PROCEADE-CRC-01 trial is currently being conducted to evaluate efficacy of Precemtabart tocentecan combined with bevacizumab ± capecitabine or bevacizumab + 5-fluorouracil(Kopetz et al., 2025a).

#### 3.3.3 ADCs combined with immunotherapy

The combination of ADCs with immune checkpoint inhibitors (ICIs) is based on a dual mechanism of “localized high-potency cytotoxicity + immune activation.” This combination treatment may potentially overcome the limitations of previous immunotherapies in CRC while enabling more refined individualized treatment. In tumours, ADCs deliver highly potent cytotoxins in a targeted manner, leading to tumour cell death and the release of tumour antigens and damage-associated molecular patterns. This promotes dendritic cell–mediated antigen presentation and T-cell activation. Moreover, ICIs (e.g., anti-PD-1/PD-L1) promote adaptive immune suppression, amplifying the ADC-induced primary immune response (Villacampa et al., 2024).

ADC + ICI strategies should be guided by multidimensional biomarkers—including molecular target expression, MSI status, and tumour-infiltrating immune phenotypes—to achieve true personalized treatment. CRC is highly heterogeneous both molecularly and immunologically, comprising MSI-H (“immune-hot”) and MSS (“immune-cold”) subtypes, different oncogenic drivers (HER2, c-MET, KRAS/BRAF, etc.), and variable antigen expression. Since the vast majority of metastatic CRCs are MSS and unresponsive to ICIs alone, ADCs may act as “tumour heaters” by inducing antigen release and recruiting effector immune cells, thereby substantially expanding the proportion of MSS patients who could benefit from ICIs (Yu et al., 2024). In syngeneic mouse tumour models, RC48 and anti–PD-1 monotherapy inhibited tumour growth. Moreover, their combination further enhanced efficacy without significant body weight loss, indicating that RC48 can sensitize tumours to immunotherapy independent of microsatellite status (Wu X. et al., 2023). Similarly, 84-EBET (a CEACAM6-targeting ADC) combined with an anti–PD-1 antibody showed marked synergy (Kogai et al., 2025).

## 4 Key challenges in ADC design and therapy

Currently employed ADCs have demonstrated increasingly favourable specificity and cytotoxic properties, highlighting their remarkable potential in cancer treatment. Nevertheless, their design and application continue to face multiple challenges, including the complexity of pharmacokinetics, inevitable toxicity, emergence of resistance and challenges in clinical translation.

### 4.1 Complex pharmacokinetics

Compared with conventional small-molecule drugs, ADCs exhibit more complex pharmacokinetic profiles. Following intravenous administration, ADCs exist in three distinct forms in circulation: the intact conjugate, the antibody resulting from linker degradation, and the free cytotoxic payload (Guo et al., 2016; Kern et al., 2016). The relative proportions of these species continuously change as the ADC binds its target antigen, undergoes internalization, and dissociates within lysosomes (Lucas et al., 2018). Typically, the concentrations of intact ADCs and naked antibodies gradually decrease over time because of internalization and antibody clearance (Malik et al., 2017). This process is modulated by the mononuclear phagocyte system and neonatal Fc receptor-mediated recycling, in which neonatal Fc receptor binds ADCs within endocytic vesicles and transports them back to the extracellular space for salvage (Xu, 2015; Hamblett et al., 2016; Mahalingaiah et al., 2019). Consequently, antibodies—including both intact ADCs and naked antibodies—generally exhibit longer half-lives than conventional small molecules do. In contrast, the free cytotoxic payload is predominantly metabolized in the liver and eliminated via urine or faeces, a process that can be affected by drug–drug interactions as well as impaired hepatic or renal function (Khera and Thurber, 2018; Mahalingaiah et al., 2019).

### 4.2 Toxicity

In accordance with the basic design principles of ADCs, the toxicity of ADCs was initially expected to be lower than that of conventional chemotherapy (Drago et al., 2021). However, most ADCs still suffer from off-target toxic effects resembling those of their cytotoxic payloads, as well as on-target toxic effects and other poorly understood, potentially life-threatening adverse events (Colombo and Rich, 2022). Currently, ADC-associated toxicity can be broadly divided into “expected” and “unexpected” categories on the basis of the adverse events typically associated with the type of payload used (Zhu et al., 2023).

#### 4.2.1 Toxicity assessment and risk management

To enhance clinical relevance, toxicity evaluation for ADCs in colorectal cancer should be reported in a standardized manner (CTCAE grading), with explicit documentation of grade ≥3 events, serious adverse events, and the frequencies of dose interruption, reduction, and discontinuation. Cross-trial comparisons should be interpreted cautiously because eligibility criteria, prior lines of therapy, baseline organ function, and supportive-care practices vary substantially across studies; these factors can inflate or attenuate apparent toxicity rates in heavily pretreated mCRC populations.

Clinically actionable toxicities can be broadly grouped into hematologic suppression, gastrointestinal toxicity, peripheral neuropathy, ocular events, hepatotoxicity, and pneumonitis/interstitial lung disease (ILD) (Mahalingaiah et al., 2019; Hackshaw et al., 2020; Powell et al., 2020). These patterns are influenced by payload class (Colombo and Rich, 2022; Zhu et al., 2023), linker stability (Donaghy, 2016), and, in some cases, target expression in normal tissues (Hu et al., 2023). A pragmatic summary of common adverse events, plausible drivers, recommended monitoring, and risk-mitigation strategies for CRC practice is provided in Table 5.

**TABLE 5:** Adverse events summary and clinical risk management considerations for ADCs in colorectal cancer.

Toxicity domain	Typical manifestations	Plausible driver(s)	High-risk factors	Monitoring	Risk management (pragmatic)
Hematologic	Neutropenia, anemia, thrombocytopenia	Payload-related marrow toxicity (e.g., Topo I inhibitors; DNA-damaging agents); systemic payload exposure	Baseline cytopenias; extensive prior chemotherapy; poor marrow reserve; liver dysfunction	CBC at baseline and each cycle; consider mid-cycle CBC early in treatment	Growth factor support per guidelines; dose delay/reduction for recurrent grade ≥3; transfusion support as needed
Gastrointestinal	Nausea/vomiting, diarrhea, mucositis, anorexia	Payload class; off-target exposure; target expression in GI tract for some antigens	Baseline diarrhea; prior irinotecan intolerance; malnutrition; dehydration; bowel obstruction risk	Baseline symptom assessment; stool frequency tracking; electrolytes if diarrhea	Early antidiarrheal/antiemetic prophylaxis; hydration; hold drug for persistent grade ≥2–3; investigate infection/obstruction
Pneumonitis/ILD	Cough, dyspnea, hypoxia; radiographic infiltrates	Class effect reported for some Topo I–based payloads; immune/inflammatory lung injury	Pre-existing ILD; chronic lung disease; prior thoracic radiation; older age; poor performance status	Baseline respiratory history ± imaging when indicated; prompt evaluation of new symptoms; consider CT scan	Immediate interruption for suspected ILD; initiate corticosteroids per severity; permanent discontinuation for confirmed grade ≥2–3
Ocular	Dry eye, blurred vision, keratopathy, conjunctivitis	Microtubule payloads and/or off-target uptake in ocular surface tissues; corneal epitheliopathy	Baseline ocular disease; contact lens use; poor tear function	Baseline ophthalmologic assessment when risk is high; symptom screening each visit	Lubricating drops; avoid contact lenses; early ophthalmology referral; dose hold/reduction for persistent grade ≥2
Peripheral neuropathy	Paresthesia, numbness, pain, motor weakness	Microtubule-disrupting payloads (e.g., auristatins, maytansinoids)	Pre-existing neuropathy (diabetes, prior oxaliplatin); older age; cumulative exposure	Baseline neurologic exam; serial symptom scoring each cycle	Dose modification based on severity; symptomatic management; consider discontinuation for progressive grade ≥2–3
Hepatotoxicity	ALT/AST elevation, hyperbilirubinemia	Systemic payload exposure; hepatic metabolism; liver tumour burden	Baseline hepatic impairment; extensive liver metastases; concomitant hepatotoxic drugs	LFTs at baseline and each cycle; monitor bilirubin closely in liver-dominant disease	Dose delay/reduction; manage drug interactions; evaluate biliary obstruction; discontinue for recurrent severe injury
Cutaneous	Rash, pruritus, hand-foot reactions (variable by platform)	Payload/off-target exposure; immune-mediated reactions	History of severe rash; atopy; concomitant targeted therapies	Skin assessment each visit; patient education for early reporting	Topical steroids/antihistamines; dose hold for grade ≥3; dermatology referral for severe or atypical cases
Infusion-related/hypersensitivity	Chills, fever, flushing, dyspnea during infusion	Antibody component; excipients; immune activation	History of infusion reactions; atopy	Vital signs during infusion; observe post-infusion in high-risk patients	Premedication when indicated; slow/interrupt infusion; treat per protocol; discontinue for severe reactions

#### 4.2.2 Mechanistic considerations by payload class and clinical implications

Across CRC-directed ADC programs, microtubule-disrupting payloads are most commonly associated with peripheral neuropathy and ocular surface events, often in a cumulative-exposure pattern (Powles et al., 2021). In contrast, Topoisomerase I inhibitor–based payloads more frequently produce myelosuppression and gastrointestinal toxicity and have also been linked to pneumonitis/ILD in some settings, underscoring the need for early recognition and protocolized intervention (Bardia et al., 2021). DNA-damaging payloads may carry broader systemic toxicity and a narrower therapeutic window, making dose optimization and patient selection particularly critical. Because mCRC patients frequently have baseline gastrointestinal symptoms, prior neurotoxic chemotherapy exposure, and substantial hepatic tumour burden, risk stratification and proactive supportive care should be integrated into trial design and routine practice to preserve treatment continuity and maximize benefit.

#### 4.2.3 Unexpected toxic effects and patient-level heterogeneity

Beyond the payload-class patterns and the clinically oriented risk-mitigation framework summarized above (Table 5), ADCs can also induce unexpected or disabling toxicities that are not readily predicted by antigen specificity alone. The mechanisms remain incompletely understood and may involve extra-tumoural antigen expression, nonspecific uptake by normal tissues, and/or systemic enzymatic cleavage resulting in exposure to deconjugated species and free payload. Historically, this phenomenon was highlighted by the LeY-targeting conjugate BR96–doxorubicin, which produced prominent gastrointestinal toxicity rather than the “expected” hematologic or cardiac toxicities typically associated with doxorubicin. (Saleh et al., 2000). Cardiotoxicity has also been observed with trastuzumab-based platforms, including T-DM1 and T-DXd, consistent with potential on-target/off-tumour liability of the antibody component, whereas such toxicity appears less common with other DXd-based programs (Hu et al., 2023).

Importantly, interstitial lung disease (ILD)/pneumonitis has been reported across multiple ADCs with variable incidence and severity and may occur with patterns that are not strictly dependent on antigen specificity or payload type, underscoring the need for early recognition and protocolized intervention (Hackshaw et al., 2020; Kumagai et al., 2020; Tarantino et al., 2021). In addition to between-drug variability, clinically meaningful heterogeneity exists among patients receiving the same ADC, as baseline organ function, comorbidities, and inter-individual PK/PD differences can modify both toxicity phenotype and severity(Powell et al., 2022; Tang M. et al., 2024); therefore, eligibility criteria, monitoring intensity, and dose-modification algorithms should be tailored accordingly. When ADCs are combined with chemotherapy or other systemic agents, these risks may be amplified through overlap or unexpected synergy, further reinforcing the value of predefined stopping rules and organ-specific management pathways.

### 4.3 Resistance

**FIGURE 2:**
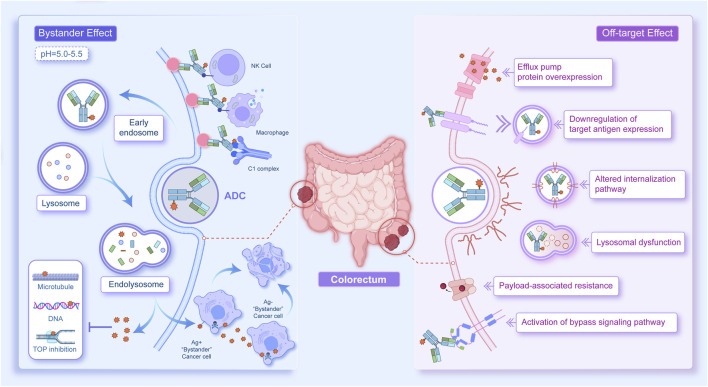
Mechanisms of action and resistance to ADCs in colorectal cancer therapy. The left panel illustrates the normal mechanism of ADC action. The antibody component of the ADC binds to the target antigen and is internalized via endocytosis, leading to invagination of the plasma membrane and formation of endosomes, which subsequently fuse with lysosomes. Following lysosomal cleavage, the payload is released into the cytoplasm, where it induces tumour cell apoptosis or death by targeting DNA breaks, disrupting microtubules, or inhibiting topoisomerases. Lipophilic payloads may also diffuse into neighbouring cells, resulting in a bystander effect. Concurrently, the monoclonal antibody component of the ADC can trigger antitumour immunity through effector functions such as ADCC, ADCP, and CDC. The right panel depicts potential mechanisms of ADC resistance, including downregulation of target antigen expression, payload resistance, impaired drug internalization and trafficking, lysosomal dysfunction, overexpression of drug efflux pumps, and activation of bypass signalling pathways.

A major challenge in ADC development is drug resistance, which can be either primary or acquired. The underlying mechanisms primarily include downregulation of target antigen expression, resistance to payloads, impaired drug internalization and trafficking, dysfunction of lysosomes, overexpression of drug efflux transporters, and activation of bypass signalling pathways (Figure 2).

#### 4.3.1 Downregulation of target antigen expression

The most common mechanism of ADC resistance is the downregulation of target antigen expression, which parallels the principles of resistance observed with monoclonal antibodies. With increasing drug exposure, target antigens may undergo mutations, copy number alterations, or structural modifications, thereby reducing the likelihood of antibody–antigen binding. Preclinical studies have demonstrated that when used to generate xenograft tumours responsive to high-dose T-DM1, JIMT1 cells resistant to trastuzumab acquire secondary resistance following cyclic T-DM1 treatment, accompanied by reduced HER2 expression (Loganzo et al., 2015; Loganzo et al., 2016). Interestingly, the bystander effect mediated by cleavable linkers can partially overcome the reduced response associated with lower antigen expression (Sung et al., 2016). In addition to decreased antigen levels, dimerization of the target antigen with another cell surface receptor can also contribute to ADC resistance. For example, NRG-1β, a ligand that induces HER2/HER3 heterodimerization, inhibits T-DM1-mediated cytotoxicity in HER2-amplified breast cancer cells. This resistance can be overcome by combination with pertuzumab, which blocks HER2/HER3 dimerization and downstream signalling (Phillips et al., 2014).

#### 4.3.2 Payload-associated resistance

Beyond aberrant antigen expression, tumour cells may also develop resistance directly to the cytotoxic payload. In the treatment of non-Hodgkin lymphoma, clinical efficacy was increased by replacing auristatin-based payloads with anthracycline-based payloads (Yu et al., 2015). In addition to payload type, the conjugation site and the average DAR are critical determinants of ADC performance (Yoder et al., 2019; Bai et al., 2020). Preclinical data indicate that while increasing the DAR enhances drug load, it also accelerates clearance, potentially reducing ADC efficacy (Sun et al., 2017).

#### 4.3.3 Impaired internalization and intracellular trafficking

ADCs are internalized into cancer cells primarily via endocytosis, which can occur through multiple pathways, including clathrin-mediated endocytosis, caveolae-mediated endocytosis, and clathrin–caveolin-independent endocytosis (Kalim et al., 2017). In a developed T-DM1-resistant model (N87-TM), upregulation of caveolin-1 (Cav-1), polymerase I, and transcriptional regulators involved in vesicle formation and endocytosis combined with reduced lysosomal colocalization impaired drug internalization and trafficking, resulting in T-DM1 insensitivity (Sung et al., 2018). In HER2-positive breast cancer, endophilin A2 promotes HER2 endocytosis and increases tumour cell sensitivity to trastuzumab. SH3GL1 knockdown in tumour cells decreases endocytosis and significantly reduces T-DM1-mediated cytotoxicity (Baldassarre et al., 2017).

#### 4.3.4 Lysosomal dysfunction

Following receptor-mediated endocytosis, ADCs are trafficked to lysosomes, where their cytotoxic payloads are released through chemical or enzymatic cleavage. Most lysosomal hydrolases require an acidic environment for optimal activity, a condition maintained by vacuolar ATPase (Mellman, 1989). This mechanism has been demonstrated in the T-DM1-resistant cell line N87-KR, which exhibited binding and internalization kinetics similar to those of parental N87 cells but showed markedly reduced lysosomal acidification due to impaired vacuolar ATPase activity. This defect diminished T-DM1 catabolism and conferred resistance (Wang et al., 2017). Beyond pH regulation, specific transporters also play critical roles, particularly for ADCs with noncleavable linkers, as their metabolites require dedicated transport proteins to reach the cytoplasm. RNA sequencing studies have identified SLC46A3 as a key transporter that mediates the cytoplasmic translocation of maytansine-based catabolites. The silencing of SLC46A3 led to the lysosomal accumulation of metabolites and ultimately drug resistance (Hamblett et al., 2015).

#### 4.3.5 Overexpression of drug efflux transporters

The overexpression of ATP-binding cassette transporters plays a pivotal role in chemotherapy resistance and has been well documented across multiple cancer types. These efflux pumps facilitate the active extrusion of drugs from tumour cells, thereby conferring an multidrug resistance phenotype (Leonard et al., 2003; Yu et al., 2013; Dong et al., 2024). Since many ADC payloads, such as maytansinoids, are substrates of ATP-binding cassette transporters, their overexpression can directly impair ADC efficacy (Kovtun et al., 2010; Cianfriglia, 2013). For instance, studies in T-DM1-resistant cells demonstrated that, despite preserving HER2 overexpression, upregulation of ABCC2 and ABCG2 promoted DM1 efflux, leading to acquired resistance. Importantly, inhibition of these transporters was shown to restore T-DM1 sensitivity (Takegawa et al., 2017).

#### 4.3.6 Activation of bypass signalling pathways

The activation of alternative signalling pathways is another key mechanism through which tumours acquire resistance to ADCs. A prototypical example is the PI3K/AKT/mTOR pathway, which regulates cell survival, growth, and metabolism. Aberrant activation of this pathway can reduce ADC sensitivity and attenuate payload efficacy. Clinically, a diminished therapeutic response to trastuzumab has been observed in patients harbouring PIK3CA mutations or with PTEN loss (Baselga et al., 2016). Mechanistic studies have indicated that PTEN deficiency or PIK3CA hyperactivation reduces trastuzumab sensitivity through constitutive activation of PI3K/AKT signalling (Berns et al., 2007). In addition, activation of the Wnt/β-catenin pathway has been implicated in ADC resistance. Overexpression of Wnt3 promotes β-catenin accumulation, thereby promoting tumour cell proliferation and invasion while simultaneously conferring resistance to trastuzumab (Wu et al., 2012).

### 4.4 Challenges in clinical translation

The complex tripartite structure of ADCs imposes stringent chemical, manufacturing, and control requirements. Major production challenges include maintaining conjugation efficiency within a batch-to-batch variability of ±5% and ensuring payload stability during the lyophilization process. These technical hurdles contribute to development costs exceeding 500 million USD for each approved ADC, with 67% of clinical-stage candidates failing because of an insufficient therapeutic index (Beck et al., 2017; Khera and Thurber, 2018; Mahalingaiah et al., 2019). Despite successful drug development, multiple challenges remain in translating ADCs into clinical practice. First, reliable biomarkers capable of predicting drug activity and toxicity are needed to guide patient selection and identify the optimal target population (Williams et al., 2020; Mathiot et al., 2024). Second, issues of high cost and economic accessibility persist. Multiple cost-effectiveness analyses have indicated that at the current prices, certain ADCs fail to meet the acceptable quality-adjusted life year gain thresholds for most healthcare systems worldwide (Liu et al., 2025; Wong et al., 2025). Finally, target homogeneity and market feasibility present further obstacles. Currently employed ADC development pipelines exhibit target redundancy, with 43% of clinical candidates directed against HER2 or TROP2 (Tarantino et al., 2022). This concentration leads to treatment duplication, while emerging targets such as CLDN6 and PTK7 are overlooked. Such market saturation may reduce commercial returns and stifle innovation in target discovery.

## 5 Future directions of ADCs: bridging innovation and personalized therapy

As CRC treatment advances towards greater precision and multidimensional development, the future trajectory of ADCs is gradually shifting from “standalone drug innovation” to “systematic and individualized solutions.” This transition encompasses not only structural innovations within ADCs—such as probody drug conjugates (PDCs) and bispecific antibodies—but also hybrid platforms integrating nanotechnology to overcome conventional delivery limitations. Moreover, rapid progress in multiomics technologies and artificial intelligence enables precise patient stratification and therapeutic response prediction, providing strong support for the application of ADCs in heterogeneous CRC populations. The integration of biomarker-driven personalized treatment strategies with novel clinical trial designs (e.g., basket/umbrella trials) further advances the transformation of ADCs from “innovative molecules” into “precision medicine tools,” thereby offering CRC patients more targeted and sustainable treatment options.

### 5.1 Innovations in ADC design

Antigen selection plays a pivotal role in determining ADC efficacy. The ideal antigen is differentially expressed between tumours and normal tissues, is located on the cell surface, and is efficiently internalized (Beck et al., 2017). With increasing research, the scope of ADC targets has expanded beyond classical tumour-associated antigens to include those in the tumour microenvironment (TME) and cancer stem cell markers (Lee et al., 2018). Computational strategies leveraging multiomics and proteomic data are accelerating antigen discovery (Schettini et al., 2021; Guan et al., 2023). In parallel, noninternalizing approaches are also under investigation, wherein ADCs target extracellular components within the TME, enabling payload release into the extracellular space and subsequent tumour penetration through diffusion and bystander effects (Ashman et al., 2022). For instance, APNU, a conjugated antibody directed against splice variants of tenascin-C, has shown complete remission in preclinical studies (Dal Corso et al., 2017). Similarly, galectin-3–binding protein, which is secreted predominantly by tumour cells, has been proposed as a potential extracellular ADC target (Giansanti et al., 2019). Therefore, the extracellular release of payloads with good diffusion and bystander effects may be a breakthrough opportunity to increase the efficacy of ADCs in the treatment of solid tumours (Staudacher and Brown, 2017).

For decades, microtubule inhibitors, topoisomerase inhibitors, and DNA-damaging agents have been the predominant payload types in ADCs. With more in-depth basic research, multiple promising strategies for payload optimization have emerged. First, the focus of payload development is gradually shifting from traditional cytotoxic drugs to novel agents with noncytotoxic mechanisms, such as topoisomerase II (Top2) inhibitors, RNA polymerase inhibitors, apoptosis inducers (e.g., Bcl-xL inhibitors), heterobifunctional protein degraders, tyrosine kinase inhibitors, and immunostimulatory agents (Beck et al., 2017; Boghaert et al., 2022; Conilh et al., 2023).

Although the development of ADCs has largely relied on classical monospecific antibodies for targeting, alternative strategies such as PDCs and biparatopic or bispecific ADCs are being actively explored. PDCs represent conditionally activated ADCs, designed by introducing a protease-cleavable masking peptide into the antigen-binding region of the antibody, which becomes selectively activated in the TME (Autio et al., 2020). In nonmalignant tissues, PDCs remain intact, but upon entry into protease-rich tumour regions, the masking peptide is cleaved, exposing the antigen-binding site and enabling the recognition of tumour antigens and release of payloads (Poreba, 2020). By delaying antigen binding and improving tumour penetration, this approach helps overcome the common “binding site barrier” in solid tumours, significantly increasing the therapeutic index and efficacy (Bordeau et al., 2021; Chen et al., 2022). Another strategy involves conjugating payloads to biparatopic or bispecific antibodies. Biparatopic antibodies bind to two distinct epitopes on the same antigen, thereby increasing binding affinity. This improved stability and tumour-targeting efficiency directly translate to superior therapeutic efficacy (Koganemaru and Shitara, 2020; Chavda et al., 2022). Bispecific antibodies, capable of targeting two different antigens, offer greater selectivity and improved tumour cell internalization, which may overcome resistance to ADCs (Mazor et al., 2015; Sellmann et al., 2016; Antonarelli et al., 2021). Several bispecific ADCs are currently under development and have entered phase I clinical trials (de Goeij et al., 2016; Andreev et al., 2017; Hong Y. et al., 2023). Moreover, the creation of trispecific and multifunctional antibodies represents a promising strategy to address receptor redundancy and tumour heterogeneity, providing new avenues for personalized treatment (Khoury et al., 2023).

### 5.2 Integration of ADCs with multidisciplinary approaches

Polymeric nanoparticles, as representative advanced drug delivery systems, are suitable carriers with multiple advantages, including enhanced drug solubility, a prolonged circulation half-life, reduced immunogenicity, controlled and targeted release, and improved safety (Ekladious et al., 2019; Patel et al., 2024; Guo et al., 2025). By exploiting the unique properties of nanoparticles, such as their small size and ability to leverage the enhanced permeability and retention effect, ADC stability can be improved, circulation time can be prolonged, and tumour targeting can be more effective (Falvo et al., 2013; El-Dakroury et al., 2024; Hong et al., 2024). In colorectal cancer models, bovine serum albumin (BSA) nanoparticles prepared by the desolvation method have been used to encapsulate small-molecule therapeutics and enhance antitumour efficacy (Kayani et al., 2018). In addition to drug encapsulation, albumin nanoparticles can serve as a carrier surface for antibody-mediated targeting; for example, an EGFR-targeted conjugate can be immobilized/adsorbed onto BSA nanoparticle surfaces to create a targeted nanocarrier with improved cellular association and antitumour activity (Ye et al., 2021). Collectively, these nanocarrier strategies highlight how albumin-based nanoparticles can be engineered for payload delivery and antibody-guided tumour targeting in CRC.

The integration of multiple types of therapies with ADCs can achieve the personalized treatment of CRC. The high molecular heterogeneity of CRC means that a single biomarker is often insufficient to predict patient responses to ADCs. With the rapid advancements in genomics, transcriptomics, proteomics, and metabolomics, researchers are now able to systematically identify molecular-level differences among CRC patients. For instance, HER2 amplification, BRAF V600E mutation, KRAS/NRAS status, and MSI are all closely associated with ADC responsiveness, providing a strong foundation for multiomics–driven precision stratification (Yaeger et al., 2018). The introduction of artificial intelligence (AI) and machine learning tools has further enabled the integration and pattern recognition of complex multiomics datasets. Using deep learning models, researchers can extract potential predictive factors of response from large-scale clinical and molecular data, thereby assisting in the identification of patients most likely to benefit (Azuaje, 2019; Kather et al., 2019). Beyond efficacy prediction, the combination of multiomics and AI also facilitates the monitoring of resistance mechanisms. By longitudinally tracking circulating tumour DNA and protein biomarkers in patient blood samples, AI algorithms can dynamically analyse tumour evolutionary trajectories, identify potential resistance mutations, and guide strategies for ADC combination or sequential therapies (Siravegna et al., 2015; Sobhani et al., 2024).

As more ADCs with innovative mechanisms enter clinical testing and combination trials progress, the development of predictive biomarkers is becoming a central focus in next-generation ADC research. Current evidence suggests that relying solely on IHC to evaluate target expression may not sufficiently predict therapeutic outcomes. To address this limitation, multiple strategies are being adopted to refine predictive models. On the one hand, new technologies, such as quantitative immunofluorescence (Moutafi et al., 2022), mass spectrometry (Zhu et al., 2020), reverse-phase protein arrays (Petricoin et al., 2023), and digital pathology–based analysis of IHC slides (Spitzmüller et al., 2023), are being employed to more precisely quantify target expression. On the other hand, the dynamic changes in target expression on circulating tumour cells are also being investigated as potential indicators for efficacy prediction (Danila et al., 2019; Pistilli et al., 2023).

### 5.3 Emerging clinical trial models

Traditional clinical trials are often stratified by tumour histology. However, given the highly complex molecular heterogeneity of CRC, this approach no longer adequately meets the needs of precision medicine. Basket and umbrella trial designs have therefore emerged. Basket trials allow the recruitment of patients across different cancer types on the basis of shared molecular markers (e.g., HER2 amplification and Trop-2 overexpression), thereby evaluating the cross-tumour applicability of specific ADCs (Hyman et al., 2018). This design is particularly important for CRC molecular subgroups with relatively small patient populations, as it helps avoid trial stagnation because of low enrolment of patients with a single cancer type. In contrast, umbrella trials stratify patients within a single cancer type (e.g., CRC) according to distinct molecular subtypes (e.g., HER2+, KRAS mutation, and CLDN18.2 expression), enabling the evaluation of multiple ADCs or ADC-based combinations in parallel (Dienstmann et al., 2017). Furthermore, adaptive trial designs combined with real-world data are accelerating the clinical translation of ADC research. By incorporating dynamic adjustment mechanisms, researchers can optimize dosing, refine patient subgroup selection, or redirect study designs in response to interim results (Berry et al., 2015). This not only increases trial efficiency but also expedites the validation and implementation of personalized treatment strategies. Collectively, these novel clinical trial models provide flexible and efficient platforms for validating ADCs in CRC, with the potential to shorten development timelines and advance the clinical practice of precision oncology.

## 6 Conclusion

As an innovative therapeutic strategy, ADCs have demonstrated significant clinical potential in a variety of solid tumours. Importantly, several ADC programs have already generated clinically meaningful signals in CRC cohorts—for example, T-DXd has shown robust activity in HER2-positive metastatic CRC in the DESTINY-CRC studies, RC48 is being evaluated in HER2-expressing advanced CRC, and CEACAM5-directed topoisomerase I inhibitor ADCs (e.g., precemtabart tocentecan/M9140) have reported encouraging disease control in heavily pretreated, irinotecan-refractory mCRC. Nevertheless, no ADC has yet been approved specifically for CRC, underscoring the substantial unmet clinical need. With continuous innovations in antibody engineering, linker design, payload diversification, and conjugation technologies, the specificity, stability, and safety of ADCs are steadily improving, enabling them to evolve into precise drug delivery systems.

The future value of ADCs lies not only in providing more treatment options but also in their deep integration with personalized medicine. Through molecular diagnostics, patient subgroups can be precisely stratified, allowing antibody selection to align with specific tumour antigen profiles. Smart linkers and adjustable DARs enable controllable payload release, while diverse payloads, such as topoisomerase inhibitors and immune modulators, can meet the therapeutic needs of patients with different molecular subtypes. Moreover, the exploration of ADCs in combination with immunotherapy, molecular targeted therapy, and chemotherapy provides new strategies to address the molecular heterogeneity and drug resistance of CRC.

In summary, ADCs constitute not only a novel form of antitumour therapy but also an advanced drug delivery system. Their integration with personalized medicine is expected to drive the overall treatment framework of “molecular diagnostics + precise payloads + combination therapy,” offering more selective and durable solutions for highly heterogeneous tumours such as CRC and ultimately reshaping the future therapeutic landscape.

## Glossary

CRC: Colorectal cancer
ADCs: Antibody–drug conjugates
mAb: Monoclonal antibody
MMAE: Monomethyl auristatin E
FDA: Food and Drug Administration
DM1: Mertansine
Nectin-4: Nectin cell adhesion molecule 4
NSCLC: Non-small-cell lung cancer
Trop-2: Trophoblast cell surface antigen-2
PBD: Pyrrolobenzodiazepine dimer
EGFR: Epidermal growth factor receptor
NMPA: National Medical Products Administration
DNA: Deoxyribonucleic acid
c-Met: Cellular-mesenchymal epithelial transition factor
IgG: Immunoglobulin G
ADCC: Antibody-dependent cellular cytotoxicity
ADCP: Antibody-dependent cellular phagocytosis
CDC: Complement-dependent cytotoxicity
**IC**_**50**_: Half maximal inhibitory concentration
HER2: Human epidermal growth factor receptor 2
ORR: Objective response rate
TME: Tumour microenvironment
BRAF: B-Raf proto-oncogene, serine/threonine kinase Gene
MSI-H: MicroSatellite instability-high
TLR: Toll like receptor
STING: Stimulator of interferon genes
RNA: Ribonucleic acid
Bcl-XL: B-cell lymphoma-extra large
DAR: Drug-to-antibody ratio
PI3K/AKT/mTOR: Phosphoinositide 3-kinase/protein kinase B/mechanistic target of rapamycin pathway
MAPK/ERK: Mitogen-activated protein kinase/extracellular signal-regulated kinase
Ras: Rat sarcoma
GSK3β: Glycogen synthase kinase 3 beta
JAK/STAT: The Janus kinase/signal transducer and activator of transcription
PD-L1: Programmed death-ligand 1
cGAS–STING: Cyclic GMP–AMP synthase–stimulator of interferon genes
IFN-β: Interferon-β
PDX: Patient-derived xenografts
CDKN1A: Cyclin dependent kinase inhibitor 1A
mCRC: Metastatic colorectal cancer
IHC: Immunohistochemistry
HER3: Epidermal growth factor receptor 3
KRAS: Kirsten ratsarcoma viral oncogene homolog
PD-1: Programmed death receptor 1
RAS/RAF/MAPK: The rat sarcoma/rapidly accelerated fibrosarcoma/mitogen-activated protein kinase
RTKs: Receptor tyrosine kinases
GCC: Guanylyl cyclase C
VC: Peptide maleimido-caproyl-valine-citrulline
CEACAM5: Carcinoembryonic antigen-related cell adhesion molecule-5
TOP1: DNA Topoisomerase I
DCR: Disease control rate
mPFS: Median progression-free survival
DLT: Dose-limiting toxicity
CDH17: Cadherin 17
MF-6: FABPs ligand 6
LGR5: Leucine-rich repeat–containing G protein–coupled receptor 5
GEMM: Genetically engineered mouse models
EBET: BET protein degrader
EREG: Epiregulin
CLDN1: Claudin-1
DR5: Death receptor 5
PDO: Patient-derived organoids
DMSA: Duocarmycin SA
CDCP1: CUB-domain containing protein 1
ADAM9: ADAM metallopeptidase domain 9
GOLPH2: Golgi phosphoprotein 2
MSS: Microsatellite stability
ICIs: Immune checkpoint inhibitors
FcRn: Neonatal Fc Receptor
NRG-1β: Recombinant human Neuregulin 1-beta
PTEN: Phosphatase and tensin homolog
Wnt3: Wingless-Type MMTV Integration Site Family, Member 3
PDCs: Probody drug conjugates
AI: Artificial intelligence

## References

[B1] Abdollahpour-Alitappeh M. Lotfinia M. Gharibi T. Mardaneh J. Farhadihosseinabadi B. Larki P. (2019). Antibody-drug conjugates (ADCs) for cancer therapy: strategies, challenges, and successes. J. Cell. Physiology 234 (5), 5628–5642. 10.1002/jcp.27419 30478951

[B2] Abu-Yousif A. O. Cvet D. Gallery M. Bannerman B. M. Ganno M. L. Smith M. D. (2020). Preclinical antitumor activity and biodistribution of a novel Anti-GCC antibody-drug conjugate in patient-derived xenografts. Mol. Cancer Ther. 19 (10), 2079–2088. 10.1158/1535-7163.MCT-19-1102 32788205

[B3] Almhanna K. Kalebic T. Cruz C. Faris J. E. Ryan D. P. Jung J. (2016). Phase I study of the investigational anti-guanylyl cyclase antibody-drug conjugate TAK-264 (MLN0264) in adult patients with advanced gastrointestinal malignancies. Clin. Cancer Res. An Official J. Am. Assoc. For Cancer Res. 22 (20), 5049–5057. 10.1158/1078-0432.CCR-15-2474 27178743

[B4] Anderson M. G. Falls H. D. Mitten M. J. Oleksijew A. Vaidya K. S. Boghaert E. R. (2020). Targeting multiple EGFR-Expressing tumors with a highly potent tumor-selective antibody-drug conjugate. Mol. Cancer Ther. 19 (10), 2117–2125. 10.1158/1535-7163.MCT-20-0149 32847977

[B5] Andreev J. Thambi N. Perez Bay A. E. Delfino F. Martin J. Kelly M. P. (2017). Bispecific antibodies and antibody-drug conjugates (ADCs) bridging HER2 and prolactin receptor improve efficacy of HER2 ADCs. Mol. Cancer Ther. 16 (4), 681–693. 10.1158/1535-7163.MCT-16-0658 28108597

[B6] Antonarelli G. Giugliano F. Corti C. Repetto M. Tarantino P. Curigliano G. (2021). Research and clinical landscape of bispecific antibodies for the treatment of solid malignancies. Pharm. Basel, Switz. 14 (9), 884. 10.3390/ph14090884 34577584 PMC8468026

[B7] Arteaga C. L. Sliwkowski M. X. Osborne C. K. Perez E. A. Puglisi F. Gianni L. (2011). Treatment of HER2-positive breast cancer: current status and future perspectives. Nat. Rev. Clin. Oncol. 9 (1), 16–32. 10.1038/nrclinonc.2011.177 22124364

[B8] Ashman N. Bargh J. D. Spring D. R. (2022). Non-internalising antibody-drug conjugates. Chem. Soc. Rev. 51 (22), 9182–9202. 10.1039/d2cs00446a 36322071

[B9] Autio K. A. Boni V. Humphrey R. W. Naing A. (2020). Probody therapeutics: an emerging class of therapies designed to enhance On-Target effects with reduced off-tumor toxicity for use in immuno-oncology. Clin. Cancer Res. An Official J. Am. Assoc. For Cancer Res. 26 (5), 984–989. 10.1158/1078-0432.CCR-19-1457 31601568 PMC8436251

[B10] Azuaje F. (2019). Artificial intelligence for precision oncology: beyond patient stratification. NPJ Precis. Oncol. 3, 6. 10.1038/s41698-019-0078-1 30820462 PMC6389974

[B11] Bai C. Reid E. E. Wilhelm A. Shizuka M. Maloney E. K. Laleau R. (2020). Site-specific conjugation of the indolinobenzodiazepine DGN549 to antibodies affords antibody-drug conjugates with an improved therapeutic index as compared with lysine conjugation. Bioconjugate Chem. 31 (1), 93–103. 10.1021/acs.bioconjchem.9b00777 31747250

[B12] Baldassarre T. Truesdell P. Craig A. W. (2017). Endophilin A2 promotes HER2 internalization and sensitivity to trastuzumab-based therapy in HER2-positive breast cancers. Breast Cancer Res. BCR 19 (1), 110. 10.1186/s13058-017-0900-z 28974266 PMC5627411

[B13] Bardia A. Hurvitz S. A. Tolaney S. M. Loirat D. Punie K. Oliveira M. (2021). Sacituzumab govitecan in metastatic triple-negative breast cancer. N. Engl. J. Med. 384 (16), 1529–1541. 10.1056/NEJMoa2028485 33882206

[B14] Bargh J. D. Isidro-Llobet A. Parker J. S. Spring D. R. (2019). Cleavable linkers in antibody-drug conjugates. Chem. Soc. Rev. 48 (16), 4361–4374. 10.1039/c8cs00676h 31294429

[B15] Barok M. Joensuu H. Isola J. (2014). Trastuzumab emtansine: mechanisms of action and drug resistance. Breast Cancer Res. BCR 16 (2), 209. 10.1186/bcr3621 24887180 PMC4058749

[B16] Baselga J. Swain S. M. (2009). Novel anticancer targets: revisiting ERBB2 and discovering ERBB3. Nat. Rev. Cancer 9 (7), 463–475. 10.1038/nrc2656 19536107

[B17] Baselga J. Lewis Phillips G. D. Verma S. Ro J. Huober J. Guardino A. E. (2016). Relationship between tumor biomarkers and efficacy in EMILIA, a phase III study of trastuzumab emtansine in HER2-Positive metastatic breast cancer. Clin. Cancer Res. An Official J. Am. Assoc. For Cancer Res. 22 (15), 3755–3763. 10.1158/1078-0432.CCR-15-2499 26920887 PMC5485412

[B18] Beauchemin N. Arabzadeh A. (2013). Carcinoembryonic antigen-related cell adhesion molecules (CEACAMs) in cancer progression and metastasis. Cancer Metastasis Rev. 32 (3), 643–671. 10.1007/s10555-013-9444-6 23903773

[B19] Beck A. Goetsch L. Dumontet C. Corvaïa N. (2017). Strategies and challenges for the next generation of antibody-drug conjugates. Nat. Rev. Drug Discov. 16 (5), 315–337. 10.1038/nrd.2016.268 28303026

[B20] Berns K. Horlings H. M. Hennessy B. T. Madiredjo M. Hijmans E. M. Beelen K. (2007). A functional genetic approach identifies the PI3K pathway as a major determinant of trastuzumab resistance in breast cancer. Cancer Cell 12 (4), 395–402. 10.1016/j.ccr.2007.08.030 17936563

[B21] Berry S. M. Connor J. T. Lewis R. J. (2015). The platform trial: an efficient strategy for evaluating multiple treatments. JAMA 313 (16), 1619–1620. 10.1001/jama.2015.2316 25799162

[B22] Boghaert E. R. Cox M. C. Vaidya K. S. (2022). Pathophysiologic and pharmacologic considerations to improve the design and application of antibody-drug conjugates. Cancer Res. 82 (10), 1858–1869. 10.1158/0008-5472.CAN-21-3236 35298624

[B23] Bokemeyer C. Bondarenko I. Hartmann J. T. de Braud F. Schuch G. Zubel A. (2011). Efficacy according to biomarker status of cetuximab plus FOLFOX-4 as first-line treatment for metastatic colorectal cancer: the OPUS study. Ann. Oncol. Official J. Eur. Soc. For Med. Oncol. 22 (7), 1535–1546. 10.1093/annonc/mdq632 21228335

[B24] Bordeau B. M. Yang Y. Balthasar J. P. (2021). Transient competitive inhibition bypasses the binding site barrier to improve tumor penetration of trastuzumab and enhance T-DM1 efficacy. Cancer Res. 81 (15), 4145–4154. 10.1158/0008-5472.CAN-20-3822 33727230 PMC8338739

[B25] Buc E. Vartanian M. D. Darcha C. Déchelotte P. Pezet D. (2005). Guanylyl cyclase C as a reliable immunohistochemical marker and its ligand *Escherichia coli* heat-stable enterotoxin as a potential protein-delivering vehicle for colorectal cancer cells. Eur. J. Cancer (Oxford, Engl. 1990) 41 (11), 1618–1627. 10.1016/j.ejca.2005.02.031 15919201

[B26] Campbell M. R. Amin D. Moasser M. M. (2010). HER3 comes of age: new insights into its functions and role in signaling, tumor biology, and cancer therapy. Clin. Cancer Res. An Official J. Am. Assoc. For Cancer Res. 16 (5), 1373–1383. 10.1158/1078-0432.CCR-09-1218 20179223 PMC2831167

[B27] Capone E. Tryggvason T. Cela I. Dufrusine B. Pinti M. Del Pizzo F. (2023). HER-3 surface expression increases in advanced colorectal cancer representing a potential therapeutic target. Cell Death Discov. 9 (1), 400. 10.1038/s41420-023-01692-8 37898642 PMC10613198

[B28] Carneiro B. A. Papadopoulos K. P. Strickler J. H. Lassman A. B. Waqar S. N. Chae Y. K. (2023). Phase I study of anti-epidermal growth factor receptor antibody-drug conjugate serclutamab talirine: safety, pharmacokinetics, and antitumor activity in advanced glioblastoma. Neuro-oncology Adv. 5 (1), vdac183. 10.1093/noajnl/vdac183 36814898 PMC9940695

[B29] Carrithers S. L. Barber M. T. Biswas S. Parkinson S. J. Park P. K. Goldstein S. D. (1996). Guanylyl cyclase C is a selective marker for metastatic colorectal tumors in human extraintestinal tissues. Proc. Natl. Acad. Sci. U. S. A. 93 (25), 14827–14832. 10.1073/pnas.93.25.14827 8962140 PMC26221

[B30] Cazes A. Betancourt O. Esparza E. Mose E. S. Jaquish D. Wong E. (2021). A MET targeting antibody-drug conjugate overcomes gemcitabine resistance in pancreatic cancer. Clin. Cancer Res. An Official J. Am. Assoc. For Cancer Res. 27 (7), 2100–2110. 10.1158/1078-0432.CCR-20-3210 33451980

[B31] Chau C. H. Steeg P. S. Figg W. D. (2019). Antibody-drug conjugates for cancer. Lancet London, Engl. 394 (10200), 793–804. 10.1016/S0140-6736(19)31774-X 31478503

[B32] Chavda V. P. Solanki H. K. Davidson M. Apostolopoulos V. Bojarska J. (2022). Peptide-drug conjugates: a new hope for cancer management. Mol. Basel, Switz. 27 (21), 7232. 10.3390/molecules27217232 36364057 PMC9658517

[B33] Chen P. Bordeau B. M. Zhang Y. Balthasar J. P. (2022). Transient inhibition of trastuzumab-tumor binding to overcome the “Binding-Site Barrier” and improve the efficacy of a trastuzumab-gelonin immunotoxin. Mol. Cancer Ther. 21 (10), 1573–1582. 10.1158/1535-7163.MCT-22-0192 35930739 PMC9547943

[B34] Cherradi S. Ayrolles-Torro A. Vezzo-Vié N. Gueguinou N. Denis V. Combes E. (2017). Antibody targeting of claudin-1 as a potential colorectal cancer therapy. J. Exp. and Clin. Cancer Res. CR 36 (1), 89. 10.1186/s13046-017-0558-5 28659146 PMC5490170

[B35] Cherradi S. Garambois V. Marines J. Andrade A. F. Fauvre A. Morand O. (2023). Improving the response to oxaliplatin by targeting chemotherapy-induced CLDN1 in resistant metastatic colorectal cancer cells. Cell and Biosci. 13 (1), 72. 10.1186/s13578-023-01015-5 37041570 PMC10091849

[B36] Chiang Z.-C. Xu S. Zhao X. Liu M. Lin J. Chen Q. (2025). Generation and characterization of 7DC-DM1: a non-cleavable CD47-targeting antibody-drug conjugates with antitumor effects. Int. J. Biol. Macromol. 310, 142844. 10.1016/j.ijbiomac.2025.142844 40187444

[B37] Chis A. A. Dobrea C. M. Arseniu A. M. Frum A. Rus L.-L. Cormos G. (2024). Antibody-drug conjugates-evolution and perspectives. Int. J. Mol. Sci. 25 (13), 6969. 10.3390/ijms25136969 39000079 PMC11241239

[B38] Choi Y. Choi Y. Hong S. (2024). Recent technological and intellectual property trends in antibody-drug conjugate research. Pharmaceutics 16 (2), 221. 10.3390/pharmaceutics16020221 38399275 PMC10892729

[B39] Chung Y.-C. Chiu H.-H. Wei W.-C. Chang K.-J. Chao W.-T. (2020). Application of trastuzumab emtansine in HER-2-positive and KRAS/BRAF-mutated colon cancer cells. Eur. J. Clin. Investigation, e13255. 10.1111/eci.13255 32350854

[B40] Cianfriglia M. (2013). The biology of MDR1-P-glycoprotein (MDR1-Pgp) in designing functional antibody drug conjugates (ADCs): the experience of gemtuzumab ozogamicin. Ann. Dell'Istituto Super. Di Sanita 49 (2), 150–168. 10.4415/ANN_13_02_07 23771260

[B41] Ciardiello F. Tortora G. (2008). EGFR antagonists in cancer treatment. N. Engl. J. Med. 358 (11), 1160–1174. 10.1056/NEJMra0707704 18337605

[B42] Colombo R. Rich J. R. (2022). The therapeutic window of antibody drug conjugates: a dogma in need of revision. Cancer Cell 40 (11), 1255–1263. 10.1016/j.ccell.2022.09.016 36240779

[B43] Conilh L. Sadilkova L. Viricel W. Dumontet C. (2023). Payload diversification: a key step in the development of antibody-drug conjugates. J. Hematol. and Oncol. 16 (1), 3. 10.1186/s13045-022-01397-y 36650546 PMC9847035

[B44] Cortés J. Diéras V. Lorenzen S. Montemurro F. Riera-Knorrenschild J. Thuss-Patience P. (2020). Efficacy and safety of trastuzumab emtansine plus capecitabine vs trastuzumab emtansine alone in patients with previously treated ERBB2 (HER2)-positive metastatic breast cancer: a phase 1 and randomized phase 2 trial. JAMA Oncol. 6 (8), 1203–1209. 10.1001/jamaoncol.2020.1796 32584367 PMC7317656

[B45] Dal Corso A. Gébleux R. Murer P. Soltermann A. Neri D. (2017). A non-internalizing antibody-drug conjugate based on an anthracycline payload displays potent therapeutic activity *in vivo* . J. Control. Release Official J. Control. Release Soc. 264, 211–218. 10.1016/j.jconrel.2017.08.040 28867376 PMC5844458

[B46] Danila D. C. Szmulewitz R. Z. Vaishampayan U. Higano C. S. Baron A. D. Gilbert H. N. (2019). Phase I study of DSTP3086S, an antibody-drug conjugate targeting six-transmembrane epithelial antigen of prostate 1, in metastatic castration-resistant prostate cancer. J. Clin. Oncol. Official J. Am. Soc. Clin. Oncol. 37 (36), 3518–3527. 10.1200/JCO.19.00646 31689155 PMC7351321

[B47] de Goeij B. E. C. G. Vink T. Ten Napel H. Breij E. C. W. Satijn D. Wubbolts R. (2016). Efficient payload delivery by a bispecific antibody-drug conjugate targeting HER2 and CD63. Mol. Cancer Ther. 15 (11), 2688–2697. 10.1158/1535-7163.MCT-16-0364 27559142

[B48] Decary S. Berne P.-F. Nicolazzi C. Lefebvre A.-M. Dabdoubi T. Cameron B. (2020). Preclinical activity of SAR408701: a novel Anti-CEACAM5-maytansinoid antibody-drug conjugate for the treatment of CEACAM5-positive epithelial tumors. Clin. Cancer Res. An Official J. Am. Assoc. For Cancer Res. 26 (24), 6589–6599. 10.1158/1078-0432.CCR-19-4051 33046521

[B49] Diamantis N. Banerji U. (2016). Antibody-drug conjugates-an emerging class of cancer treatment. Br. J. Cancer 114 (4), 362–367. 10.1038/bjc.2015.435 26742008 PMC4815767

[B50] Díaz-Serrano A. Angulo B. Dominguez C. Pazo-Cid R. Salud A. Jiménez-Fonseca P. (2018). Genomic profiling of HER2-Positive gastric cancer: PI3K/Akt/mTOR pathway as predictor of outcomes in HER2-Positive advanced gastric cancer treated with trastuzumab. Oncol. 23 (9), 1092–1102. 10.1634/theoncologist.2017-0379 29700210 PMC6192610

[B51] Dienstmann R. Vermeulen L. Guinney J. Kopetz S. Tejpar S. Tabernero J. (2017). Consensus molecular subtypes and the evolution of precision medicine in colorectal cancer. Nat. Rev. Cancer 17 (2), 79–92. 10.1038/nrc.2016.126 28050011

[B52] Ding J. Liu Z. Liu S. Xie X. Yin Q. Lu W. (2025). Preparation and anti-tumor ability evaluation of anti-PD-L1 conjugated curcumin in colon cancer. Int. J. Biol. Macromol. 306 (Pt 3), 141563. 10.1016/j.ijbiomac.2025.141563 40037453

[B53] Donaghy H. (2016). Effects of antibody, drug and linker on the preclinical and clinical toxicities of antibody-drug conjugates. MAbs 8 (4), 659–671. 10.1080/19420862.2016.1156829 27045800 PMC4966843

[B54] Dong W. Shi J. Yuan T. Qi B. Yu J. Dai J. (2019). Antibody-drug conjugates of 7-ethyl-10-hydroxycamptothecin: Sacituzumab govitecan and labetuzumab govitecan. Eur. J. Med. Chem. 167, 583–593. 10.1016/j.ejmech.2019.02.017 30822636

[B55] Dong X.-D. Lu Q. Li Y.-D. Cai C.-Y. Teng Q.-X. Lei Z.-N. (2024). RN486, a Bruton's tyrosine kinase inhibitor, antagonizes multidrug resistance in ABCG2-overexpressing cancer cells. J. Transl. Intern. Med. 12 (3), 288–298. 10.2478/jtim-2024-0011 39081282 PMC11284896

[B56] Dotan E. Cohen S. J. Starodub A. N. Lieu C. H. Messersmith W. A. Simpson P. S. (2017). Phase I/II trial of labetuzumab govitecan (Anti-CEACAM5/SN-38 antibody-drug conjugate) in patients with refractory or relapsing metastatic colorectal cancer. J. Clin. Oncol. Official J. Am. Soc. Clin. Oncol. 35 (29), 3338–3346. 10.1200/JCO.2017.73.9011 28817371 PMC8259133

[B57] Douillard J. Y. Siena S. Cassidy J. Tabernero J. Burkes R. Barugel M. (2014). Final results from PRIME: randomized phase III study of panitumumab with FOLFOX4 for first-line treatment of metastatic colorectal cancer. Ann. Oncol. Official J. Eur. Soc. For Med. Oncol. 25 (7), 1346–1355. 10.1093/annonc/mdu141 24718886

[B58] Drago J. Z. Modi S. Chandarlapaty S. (2021). Unlocking the potential of antibody-drug conjugates for cancer therapy. Nat. Rev. Clin. Oncol. 18 (6), 327–344. 10.1038/s41571-021-00470-8 33558752 PMC8287784

[B59] Dumontet C. Reichert J. M. Senter P. D. Lambert J. M. Beck A. (2023). Antibody-drug conjugates come of age in oncology. Nat. Rev. Drug Discov. 22 (8), 641–661. 10.1038/s41573-023-00709-2 37308581

[B60] Dyson M. R. Masters E. Pazeraitis D. Perera R. L. Syrjanen J. L. Surade S. (2020). Beyond affinity: selection of antibody variants with optimal biophysical properties and reduced immunogenicity from mammalian display libraries. MAbs 12 (1), 1829335. 10.1080/19420862.2020.1829335 33103593 PMC7592150

[B61] Eades-Perner A. M. van der Putten H. Hirth A. Thompson J. Neumaier M. von Kleist S. (1994). Mice transgenic for the human carcinoembryonic antigen gene maintain its spatiotemporal expression pattern. Cancer Res. 54 (15), 4169–4176. 8033149

[B62] Ekladious I. Colson Y. L. Grinstaff M. W. (2019). Polymer-drug conjugate therapeutics: advances, insights and prospects. Nat. Rev. Drug Discov. 18 (4), 273–294. 10.1038/s41573-018-0005-0 30542076 PMC12032968

[B63] El-Dakroury W. A. Zewail M. B. Asaad G. F. Abdallah H. M. I. Shabana M. E. Said A. R. (2024). Fexofenadine-loaded chitosan coated solid lipid nanoparticles (SLNs): a potential oral therapy for ulcerative colitis. Eur. J. Pharm. Biopharm. Official J. Arbeitsgemeinschaft Fur Pharmazeutische Verfahrenstechnik E.V 196, 114205. 10.1016/j.ejpb.2024.114205 38311187

[B64] Falvo E. Tremante E. Fraioli R. Leonetti C. Zamparelli C. Boffi A. (2013). Antibody-drug conjugates: targeting melanoma with cisplatin encapsulated in protein-cage nanoparticles based on human ferritin. Nanoscale 5 (24), 12278–12285. 10.1039/c3nr04268e 24150593

[B65] Feng L. Yao H.-P. Wang W. Zhou Y.-Q. Zhou J. Zhang R. (2014). Efficacy of Anti-RON antibody Zt/g4–Drug maytansinoid conjugation (Anti-RON ADC) as a novel therapeutics for targeted colorectal cancer therapy. Clin. Cancer Res. 20 (23), 6045–6058. 10.1158/1078-0432.Ccr-14-0898 25294907

[B66] Fu Z. Li S. Han S. Shi C. Zhang Y. (2022). Antibody drug conjugate: the “biological missile” for targeted cancer therapy. Signal Transduct. Target. Ther. 7 (1), 93. 10.1038/s41392-022-00947-7 35318309 PMC8941077

[B67] Gallery M. Zhang J. Bradley D. P. Brauer P. Cvet D. Estevam J. (2018). A monomethyl auristatin E-conjugated antibody to guanylyl cyclase C is cytotoxic to target-expressing cells *in vitro* and *in vivo* . PloS One 13 (1), e0191046. 10.1371/journal.pone.0191046 29370189 PMC5784926

[B68] Ganesh K. Stadler Z. K. Cercek A. Mendelsohn R. B. Shia J. Segal N. H. (2019). Immunotherapy in colorectal cancer: rationale, challenges and potential. Nat. Rev. Gastroenterol. Hepatol. 16 (6), 361–375. 10.1038/s41575-019-0126-x 30886395 PMC7295073

[B69] Gazzah A. Bedard P. L. Hierro C. Kang Y. K. Abdul Razak A. Ryu M. H. (2022). Safety, pharmacokinetics, and antitumor activity of the anti-CEACAM5-DM4 antibody-drug conjugate tusamitamab ravtansine (SAR408701) in patients with advanced solid tumors: first-in-human dose-escalation study. Ann. Oncol. Official J. Eur. Soc. For Med. Oncol. 33 (4), 416–425. 10.1016/j.annonc.2021.12.012 35026412

[B70] Giansanti F. Capone E. Ponziani S. Piccolo E. Gentile R. Lamolinara A. (2019). Secreted Gal-3BP is a novel promising target for non-internalizing antibody-drug conjugates. J. Control. Release Official J. Control. Release Soc. 294, 176–184. 10.1016/j.jconrel.2018.12.018 30553852

[B71] Glaviano A. Foo A. S. C. Lam H. Y. Yap K. C. H. Jacot W. Jones R. H. (2023). PI3K/AKT/mTOR signaling transduction pathway and targeted therapies in cancer. Mol. Cancer 22 (1), 138. 10.1186/s12943-023-01827-6 37596643 PMC10436543

[B72] Gong X. Azhdarinia A. Ghosh S. C. Xiong W. An Z. Liu Q. (2016). LGR5-Targeted antibody-drug conjugate eradicates gastrointestinal tumors and prevents recurrence. Mol. Cancer Ther. 15 (7), 1580–1590. 10.1158/1535-7163.MCT-16-0114 27207778

[B73] Govindan S. V. Cardillo T. M. Moon S.-J. Hansen H. J. Goldenberg D. M. (2009). CEACAM5-targeted therapy of human colonic and pancreatic cancer xenografts with potent labetuzumab-SN-38 immunoconjugates. Clin. Cancer Res. An Official J. Am. Assoc. For Cancer Res. 15 (19), 6052–6061. 10.1158/1078-0432.CCR-09-0586 19789330 PMC2769088

[B74] Govindan S. V. Cardillo T. M. Rossi E. A. Trisal P. McBride W. J. Sharkey R. M. (2015). Improving the therapeutic index in cancer therapy by using antibody-drug conjugates designed with a moderately cytotoxic drug. Mol. Pharm. 12 (6), 1836–1847. 10.1021/mp5006195 25402018

[B75] Grairi M. Le Borgne M. (2024). Antibody–drug conjugates: prospects for the next generation. Drug Discov. Today 29 (12), 104241. 10.1016/j.drudis.2024.104241 39542204

[B76] Guan H. Wu Y. Li L. U. Yang Y. Qiu S. Zhao Z. (2023). Tumor neoantigens: novel strategies for application of cancer immunotherapy. Oncol. Res. 31 (4), 437–448. 10.32604/or.2023.029924 37415744 PMC10319592

[B77] Guastalla J. P. Diéras V. (2003). The taxanes: toxicity and quality of life considerations in advanced ovarian cancer. Br. J. Cancer 89 (Suppl. 3), S16–S22. 10.1038/sj.bjc.6601496 14661042 PMC2750618

[B78] Guinney J. Dienstmann R. Wang X. de Reyniès A. Schlicker A. Soneson C. (2015). The consensus molecular subtypes of colorectal cancer. Nat. Med. 21 (11), 1350–1356. 10.1038/nm.3967 26457759 PMC4636487

[B79] Guo J. Kumar S. Chipley M. Marcq O. Gupta D. Jin Z. (2016). Characterization and higher-order structure assessment of an interchain cysteine-based ADC: impact of drug loading and distribution on the mechanism of aggregation. Bioconjugate Chem. 27 (3), 604–615. 10.1021/acs.bioconjchem.5b00603 26829368

[B80] Guo Y. Huang J. Lin M. Yin Q. Zhang T. Guo Z. (2025). Nano particle loaded EZH2 inhibitors: increased efficiency and reduced toxicity for malignant solid tumors. J. Transl. Intern. Med. 13 (2), 156–169. 10.1515/jtim-2025-0020 40443399 PMC12116265

[B81] Gymnopoulos M. Betancourt O. Blot V. Fujita R. Galvan D. Lieuw V. (2020). TR1801-ADC: a highly potent cMet antibody-drug conjugate with high activity in patient-derived xenograft models of solid tumors. Mol. Oncol. 14 (1), 54–68. 10.1002/1878-0261.12600 31736230 PMC6944112

[B82] Hackshaw M. D. Danysh H. E. Singh J. Ritchey M. E. Ladner A. Taitt C. (2020). Incidence of pneumonitis/interstitial lung disease induced by HER2-targeting therapy for HER2-positive metastatic breast cancer. Breast Cancer Res. Treat. 183 (1), 23–39. 10.1007/s10549-020-05754-8 32591987 PMC7376509

[B83] Haikala H. M. Jänne P. A. (2021). Thirty years of HER3: from basic biology to therapeutic interventions. Clin. Cancer Res. An Official J. Am. Assoc. For Cancer Res. 27 (13), 3528–3539. 10.1158/1078-0432.CCR-20-4465 33608318 PMC8254743

[B84] Haikala H. M. Lopez T. Köhler J. Eser P. O. Xu M. Zeng Q. (2022). EGFR inhibition enhances the cellular uptake and antitumor-activity of the HER3 antibody-drug conjugate HER3-DXd. Cancer Res. 82 (1), 130–141. 10.1158/0008-5472.CAN-21-2426 34548332 PMC8732289

[B85] Hamblett K. J. Jacob A. P. Gurgel J. L. Tometsko M. E. Rock B. M. Patel S. K. (2015). SLC46A3 is required to transport catabolites of noncleavable antibody maytansine conjugates from the lysosome to the cytoplasm. Cancer Res. 75 (24), 5329–5340. 10.1158/0008-5472.CAN-15-1610 26631267

[B86] Hamblett K. J. Le T. Rock B. M. Rock D. A. Siu S. Huard J. N. (2016). Altering antibody-drug conjugate binding to the neonatal Fc receptor impacts efficacy and tolerability. Mol. Pharm. 13 (7), 2387–2396. 10.1021/acs.molpharmaceut.6b00153 27248573

[B87] Han S. Shin H. Lee J.-K. Liu Z. Rabadan R. Lee J. (2019). Secretome analysis of patient-derived GBM tumor spheres identifies midkine as a potent therapeutic target. Exp. and Mol. Med. 51 (12), 1–11. 10.1038/s12276-019-0351-y 31811117 PMC6897967

[B88] Han C. J. Ning X. Burd C. E. Spakowicz D. J. Tounkara F. Kalady M. F. (2024). Chemotoxicity and associated risk factors in colorectal cancer: a systematic review and meta-analysis. Cancers (Basel) 16 (14), 2597. 10.3390/cancers16142597 39061235 PMC11274507

[B89] Haratani K. Yonesaka K. Takamura S. Maenishi O. Kato R. Takegawa N. (2020). U3-1402 sensitizes HER3-expressing tumors to PD-1 blockade by immune activation. J. Clin. Investigation 130 (1), 374–388. 10.1172/JCI126598 31661465 PMC6934205

[B90] Hashimoto Y. Koyama K. Kamai Y. Hirotani K. Ogitani Y. Zembutsu A. (2019). A novel HER3-Targeting antibody-drug conjugate, U3-1402, exhibits potent therapeutic efficacy through the delivery of cytotoxic payload by efficient internalization. Clin. Cancer Res. An Official J. Am. Assoc. For Cancer Res. 25 (23), 7151–7161. 10.1158/1078-0432.CCR-19-1745 31471314

[B91] Hassan G. Seno M. (2022). ERBB signaling pathway in cancer stem cells. Adv. Exp. Med. Biol. 1393, 65–81. 10.1007/978-3-031-12974-2_3 36587302

[B92] Hock M. B. Thudium K. E. Carrasco-Triguero M. Schwabe N. F. (2015). Immunogenicity of antibody drug conjugates: bioanalytical methods and monitoring strategy for a novel therapeutic modality. AAPS J. 17 (1), 35–43. 10.1208/s12248-014-9684-6 25380723 PMC4287284

[B93] Hong X. Chen X. Wang H. Xu Q. Xiao K. Zhang Y. (2023a). A HER2-targeted antibody-drug conjugate, RC48-ADC, exerted promising antitumor efficacy and safety with intravesical instillation in preclinical models of bladder cancer. Adv. Sci. Weinheim, Baden-Wurttemberg, Ger. 10 (32), e2302377. 10.1002/advs.202302377 37824205 PMC10646285

[B94] Hong Y. Nam S.-M. Moon A. (2023b). Antibody-drug conjugates and bispecific antibodies targeting cancers: applications of click chemistry. Archives Pharmacal Res. 46 (3), 131–148. 10.1007/s12272-023-01433-6 36877356

[B95] Hong J. Li K. He J. Liang M. (2024). A new age of drug delivery: a comparative perspective of ferritin-drug conjugates (FDCs) and antibody-drug conjugates (ADCs). Bioconjugate Chem. 35 (8), 1142–1147. 10.1021/acs.bioconjchem.4c00254 39129506

[B96] Hu Y. Zu C. Zhang M. Wei G. Li W. Fu S. (2023). Safety and efficacy of CRISPR-Based non-viral PD1 locus specifically integrated anti-CD19 CAR-T cells in patients with relapsed or refractory non-hodgkin's lymphoma: a first-in-human phase I study. EClinicalMedicine 60, 102010. 10.1016/j.eclinm.2023.102010 37251628 PMC10209187

[B97] Hyman D. M. Piha-Paul S. A. Won H. Rodon J. Saura C. Shapiro G. I. (2018). HER kinase inhibition in patients with HER2-and HER3-mutant cancers. Nature 554 (7691), 189–194. 10.1038/nature25475 29420467 PMC5808581

[B98] Hynes N. E. Lane H. A. (2005). ERBB receptors and cancer: the complexity of targeted inhibitors. Nat. Rev. Cancer 5 (5), 341–354. 10.1038/nrc1609 15864276

[B99] Jacob J. Francisco L. E. Chatterjee T. Liang Z. Subramanian S. Liu Q. J. (2023). An antibody-drug conjugate targeting GPR56 demonstrates efficacy in preclinical models of colorectal cancer. Br. J. Cancer 128 (8), 1592–1602. 10.1038/s41416-023-02192-3 36759728 PMC10070492

[B100] Jacob J. Anami Y. High P. C. Liang Z. Subramanian S. Ghosh S. C. (2025). Antibody-drug conjugates targeting the EGFR ligand epiregulin elicit robust antitumor activity in colorectal cancer. Cancer Res. 85 (5), 973–986. 10.1158/0008-5472.CAN-24-0798 39693606 PMC11875910

[B101] Jeng K.-S. Jeng C.-J. Sheen I. S. Wu S.-H. Lu S.-J. Wang C.-H. (2018). Glioma-associated oncogene homolog inhibitors have the potential of suppressing cancer stem cells of breast cancer. Int. J. Mol. Sci. 19 (5). 10.3390/ijms19051375 29734730 PMC5983844

[B102] Jiang L. Zhao X. Li Y. Hu Y. Sun Y. Liu S. (2024). The tumor immune microenvironment remodeling and response to HER2-targeted therapy in HER2-positive advanced gastric cancer. IUBMB Life 76 (7), 420–436. 10.1002/iub.2804 38126920

[B103] Jin Y. Schladetsch M. A. Huang X. Balunas M. J. Wiemer A. J. (2022). Stepping forward in antibody-drug conjugate development. Pharmacol. and Ther. 229, 107917. 10.1016/j.pharmthera.2021.107917 34171334 PMC8702582

[B104] Jing X. Luo Z. Wu J. Ye F. Li J. Song Z. (2023). The genomic and immune landscapes of gastric cancer and their correlations with HER2 amplification and PD-L1 expression. Cancer Med. 12 (24), 21905–21919. 10.1002/cam4.6765 38050871 PMC10757096

[B105] Jones H. G. Jenkins G. Williams N. Griffiths P. Chambers P. Beynon J. (2017). Genetic and epigenetic intra-tumour heterogeneity in colorectal cancer. World J. Surg. 41 (5), 1375–1383. 10.1007/s00268-016-3860-z 28097409 PMC5394146

[B106] Joshi S. S. Badgwell B. D. (2021). Current treatment and recent progress in gastric cancer. CA A Cancer J. For Clin. 71 (3), 264–279. 10.3322/caac.21657 33592120 PMC9927927

[B107] Joto N. Ishii M. Minami M. Kuga H. Mitsui I. Tohgo A. (1997). DX-8951f, a water-soluble camptothecin analog, exhibits potent antitumor activity against a human lung cancer cell line and its SN-38-resistant variant. Int. J. Cancer 72 (4), 680–686. 10.1002/(sici)1097-0215(19970807)72:4<680::aid-ijc21>3.0.co;2-e 9259410

[B108] Junttila M. R. Mao W. Wang X. Wang B.-E. Pham T. Flygare J. (2015). Targeting LGR5+ cells with an antibody-drug conjugate for the treatment of colon cancer. Sci. Transl. Med. 7 (314), 314ra186. 10.1126/scitranslmed.aac7433 26582901

[B109] Kalim M. Chen J. Wang S. Lin C. Ullah S. Liang K. (2017). Intracellular trafficking of new anticancer therapeutics: antibody-drug conjugates. Drug Des. Dev. Ther. 11, 2265–2276. 10.2147/DDDT.S135571 28814834 PMC5546728

[B110] Kather J. N. Krisam J. Charoentong P. Luedde T. Herpel E. Weis C.-A. (2019). Predicting survival from colorectal cancer histology slides using deep learning: a retrospective multicenter study. PLoS Med. 16 (1), e1002730. 10.1371/journal.pmed.1002730 30677016 PMC6345440

[B111] Kayani Z. Firuzi O. Bordbar A.-K. (2018). Doughnut-shaped bovine serum albumin nanoparticles loaded with doxorubicin for overcoming multidrug-resistant in cancer cells. Int. J. Biol. Macromol. 107 (Pt B), 1835–1843. 10.1016/j.ijbiomac.2017.10.041 29030194

[B112] Keam S. J. (2020). Trastuzumab deruxtecan: first approval. Drugs 80 (5), 501–508. 10.1007/s40265-020-01281-4 32144719

[B113] Kelly R. K. Olson D. L. Sun Y. Wen D. Wortham K. A. Antognetti G. (2011). An antibody-cytotoxic conjugate, BIIB015, is a new targeted therapy for Cripto positive tumours. Eur. J. Cancer (Oxford, Engl. 1990) 47 (11), 1736–1746. 10.1016/j.ejca.2011.02.023 21458984

[B114] Kern J. C. Dooney D. Zhang R. Liang L. Brandish P. E. Cheng M. (2016). Novel phosphate modified cathepsin B linkers: improving aqueous solubility and enhancing payload scope of ADCs. Bioconjugate Chem. 27 (9), 2081–2088. 10.1021/acs.bioconjchem.6b00337 27469406

[B115] Khan T. Lyons N. J. Gough M. Kwah K. K. X. Cuda T. J. Snell C. E. (2022). CUB domain-containing protein 1 (CDCP1) is a rational target for the development of imaging tracers and antibody-drug conjugates for cancer detection and therapy. Theranostics 12 (16), 6915–6930. 10.7150/thno.78171 36276654 PMC9576610

[B116] Khera E. Thurber G. M. (2018). Pharmacokinetic and immunological considerations for expanding the therapeutic window of next-generation antibody-drug conjugates. BioDrugs Clin. Immunother. Biopharm. Gene Ther. 32 (5), 465–480. 10.1007/s40259-018-0302-5 30132210

[B117] Khongorzul P. Ling C. J. Khan F. U. Ihsan A. U. Zhang J. (2020). Antibody-drug conjugates: a comprehensive review. Mol. Cancer Res. MCR 18 (1), 3–19. 10.1158/1541-7786.MCR-19-0582 31659006

[B118] Khoury R. Saleh K. Khalife N. Saleh M. Chahine C. Ibrahim R. (2023). Mechanisms of resistance to antibody-drug conjugates. Int. J. Mol. Sci. 24 (11), 9674. 10.3390/ijms24119674 37298631 PMC10253543

[B119] Kim R. Leal A. D. Parikh A. Ryan D. P. Wang S. Bahamon B. (2023a). A phase I, first-in-human study of TAK-164, an antibody-drug conjugate, in patients with advanced gastrointestinal cancers expressing guanylyl cyclase C. Cancer Chemother. Pharmacol. 91 (4), 291–300. 10.1007/s00280-023-04507-w 36738333 PMC10068631

[B120] Kim Y. Bae Y. J. Kim J.-H. Kim H. Shin S.-J. Jung D. H. (2023b). Wnt/β-catenin pathway is a key signaling pathway to trastuzumab resistance in gastric cancer cells. BMC Cancer 23 (1), 922. 10.1186/s12885-023-11447-4 37773114 PMC10542239

[B121] Kogai H. Tsukamoto S. Koga M. Miyano M. Akagi T. Yamaguchi A. (2025). Broad-spectrum efficacy of CEACAM6-Targeted antibody-drug conjugate with BET protein degrader in colorectal, lung, and breast cancer mouse models. Mol. Cancer Ther. 24 (3), 392–405. 10.1158/1535-7163.MCT-24-0444 39812376 PMC11876960

[B122] Koganemaru S. Shitara K. (2020). Antibody-drug conjugates to treat gastric cancer. Expert Opin. Biol. Ther. 21 (7), 923–930. 10.1080/14712598.2020.1802423 32713216

[B123] Koganemaru S. Kuboki Y. Koga Y. Kojima T. Yamauchi M. Maeda N. (2019). U3-1402, a novel HER3-Targeting antibody-drug conjugate, for the treatment of colorectal cancer. Mol. Cancer Ther. 18 (11), 2043–2050. 10.1158/1535-7163.MCT-19-0452 31395690

[B124] Kopetz S. Boni V. Kato K. Raghav K. P. S. Vieito M. Pallis A. (2025a). Precemtabart tocentecan, an anti-CEACAM5 antibody–drug conjugate, in metastatic colorectal cancer: a phase 1 trial. Nat. Med. 31, 3504–3513. 10.1038/s41591-025-03843-z 40739424 PMC12532702

[B125] Kopetz S. Garcia-Carbonero R. Han S.-W. Rodriguez Rivera I. I. Ruffinelli J. C. Boni V. (2025b). Precemtabart tocentecan (M9140), an anti-CEACAM5 ADC with exatecan payload, in patients with metastatic colorectal cancer (mCRC): results from the dose optimization of the phase 1 PROCEADE CRC-01 study. J. Clin. Oncol. 43 (16_Suppl. l), 3038. 10.1200/JCO.2025.43.16_suppl.3038

[B126] Kovtun Y. V. Audette C. A. Ye Y. Xie H. Ruberti M. F. Phinney S. J. (2006). Antibody-drug conjugates designed to eradicate tumors with homogeneous and heterogeneous expression of the target antigen. Cancer Res. 66 (6), 3214–3221. 10.1158/0008-5472.CAN-05-3973 16540673

[B127] Kovtun Y. V. Audette C. A. Mayo M. F. Jones G. E. Doherty H. Maloney E. K. (2010). Antibody-maytansinoid conjugates designed to bypass multidrug resistance. Cancer Res. 70 (6), 2528–2537. 10.1158/0008-5472.CAN-09-3546 20197459

[B128] Kroemer G. Galassi C. Zitvogel L. Galluzzi L. (2022). Immunogenic cell stress and death. Nat. Immunol. 23 (4), 487–500. 10.1038/s41590-022-01132-2 35145297

[B129] Krop I. E. Masuda N. Mukohara T. Takahashi S. Nakayama T. Inoue K. (2023). Patritumab deruxtecan (HER3-DXd), a human epidermal growth factor receptor 3-Directed antibody-drug conjugate, in patients with previously treated human epidermal growth factor receptor 3-Expressing metastatic breast cancer: a multicenter, phase I/II trial. J. Clin. Oncol. Official J. Am. Soc. Clin. Oncol. 41 (36), 5550–5560. 10.1200/JCO.23.00882 37801674 PMC10730028

[B130] Kumagai K. Aida T. Tsuchiya Y. Kishino Y. Kai K. Mori K. (2020). Interstitial pneumonitis related to trastuzumab deruxtecan, a human epidermal growth factor receptor 2-targeting Ab-drug conjugate, in monkeys. Cancer Sci. 111 (12), 4636–4645. 10.1111/cas.14686 33051938 PMC7734153

[B131] Kumagai S. Koyama S. Nishikawa H. (2021). Antitumour immunity regulated by aberrant ERBB family signalling. Nat. Rev. Cancer 21 (3), 181–197. 10.1038/s41568-020-00322-0 33462501

[B132] La Monica S. Cretella D. Bonelli M. Fumarola C. Cavazzoni A. Digiacomo G. (2017). Trastuzumab emtansine delays and overcomes resistance to the third-generation EGFR-TKI osimertinib in NSCLC EGFR mutated cell lines. J. Exp. and Clin. Cancer Res. CR 36 (1), 174. 10.1186/s13046-017-0653-7 29202823 PMC5716361

[B133] La Salvia A. Lopez-Gomez V. Garcia-Carbonero R. (2019). HER2-targeted therapy: an emerging strategy in advanced colorectal cancer. Expert Opin. Investigational Drugs 28 (1), 29–38. 10.1080/13543784.2019.1555583 30513002

[B134] Lee Y. T. Tan Y. J. Oon C. E. (2018). Molecular targeted therapy: treating cancer with specificity. Eur. J. Pharmacol. 834, 188–196. 10.1016/j.ejphar.2018.07.034 30031797

[B135] Leonard G. D. Fojo T. Bates S. E. (2003). The role of ABC transporters in clinical practice. Oncol. 8 (5), 411–424. 10.1634/theoncologist.8-5-411 14530494

[B136] Li F. Emmerton K. K. Jonas M. Zhang X. Miyamoto J. B. Setter J. R. (2016a). Intracellular released payload influences potency and bystander-killing effects of antibody-drug conjugates in preclinical models. Cancer Res. 76 (9), 2710–2719. 10.1158/0008-5472.CAN-15-1795 26921341

[B137] Li H. Yu C. Jiang J. Huang C. Yao X. Xu Q. (2016b). An anti-HER2 antibody conjugated with monomethyl auristatin E is highly effective in HER2-positive human gastric cancer. Cancer Biol. and Ther. 17 (4), 346–354. 10.1080/15384047.2016.1139248 26853765 PMC4910924

[B138] Li M. Zhao X. Yu C. Wang L. (2024a). Antibody-drug conjugate overview: a state-of-the-art manufacturing process and control strategy. Pharm. Res. 41 (3), 419–440. 10.1007/s11095-023-03649-z 38366236

[B139] Li Q. Geng S. Luo H. Wang W. Mo Y. Q. Luo Q. (2024b). Signaling pathways involved in colorectal cancer: pathogenesis and targeted therapy. Signal Transduct. Target Ther. 9 (1), 266. 10.1038/s41392-024-01953-7 39370455 PMC11456611

[B140] Li X. Yao J. Qu C. Luo L. Li B. Zhang Y. (2024c). DB-1310, an ADC comprised of a novel anti-HER3 antibody conjugated to a DNA topoisomerase I inhibitor, is highly effective for the treatment of HER3-positive solid tumors. J. Transl. Med. 22 (1), 362. 10.1186/s12967-024-05133-7 38632563 PMC11022355

[B141] Li Z. Song Z. Hong W. Yang N. Wang Y. Jian H. (2024d). SHR-A1811 (antibody-drug conjugate) in advanced HER2-mutant non-small cell lung cancer: a multicenter, open-label, phase 1/2 study. Signal Transduct. Target. Ther. 9 (1), 182. 10.1038/s41392-024-01897-y 39004647 PMC11247081

[B142] Li J. J. Wang Z. H. Chen L. Zhang W. J. Ma L. X. X. Wu J. (2025a). Efficacy and safety of neoadjuvant SHR-A1811 with or without pyrotinib in women with locally advanced or early HER2-positive breast cancer: a randomized, open-label, phase II trial. Ann. Oncol. Official J. Eur. Soc. For Med. Oncol. 36 (6), 651–659. 10.1016/j.annonc.2025.02.011 40049447

[B143] Li X. Liang L. Zhu Z. Hua H. Qiu Y. (2025b). DB-1310, a HER3-targeting antibody-drug conjugate, has synergistic anti-tumor activity with trastuzumab in HER2-and HER3-expressing breast cancer. Cancer Biol. and Med. 22 (3), 231–236. 10.20892/j.issn.2095-3941.2024.0586 40110648 PMC11976708

[B144] Liewen H. Markuly N. Läubli H. Liu Y. Matter M. S. Liewen N. (2019). Therapeutic targeting of golgi phosphoprotein 2 (GOLPH2) with armed antibodies: a preclinical study of Anti-GOLPH2 antibody drug conjugates in lung and colorectal cancer models of patient derived xenografts (PDX). Target. Oncol. 14 (5), 577–590. 10.1007/s11523-019-00667-z 31541350

[B145] Linnekamp J. F. Wang X. Medema J. P. Vermeulen L. (2015). Colorectal cancer heterogeneity and targeted therapy: a case for molecular disease subtypes. Cancer Res. 75 (2), 245–249. 10.1158/0008-5472.CAN-14-2240 25593032

[B146] Liu W. Yuan J. Liu Z. Zhang J. Chang J. (2018). Label-free quantitative proteomics combined with biological validation reveals activation of Wnt/β-Catenin pathway contributing to trastuzumab resistance in gastric cancer. Int. J. Mol. Sci. 19 (7). 10.3390/ijms19071981 29986466 PMC6073113

[B147] Liu H. Zhou D. Liu D. Xu X. Zhang K. Hu R. (2024). Synergistic antitumor activity between HER2 antibody-drug conjugate and chemotherapy for treating advanced colorectal cancer. Cell Death and Dis. 15 (3), 187. 10.1038/s41419-024-06572-2 38443386 PMC10914798

[B148] Liu S. Wang K. Chen H. Wan Z. Dou L. Li S. (2025). Cost-effectiveness of sacituzumab govitecan for hormone receptor-positive human epidermal growth factor receptor 2-negative metastatic breast cancer based on the EVER-132-002 trial in China. Cost Eff. Resour. Allocation 23 (1), 8. 10.1186/s12962-025-00613-z 40108623 PMC11924825

[B149] Loganzo F. Tan X. Sung M. Jin G. Myers J. S. Melamud E. (2015). Tumor cells chronically treated with a trastuzumab-maytansinoid antibody-drug conjugate develop varied resistance mechanisms but respond to alternate treatments. Mol. Cancer Ther. 14 (4), 952–963. 10.1158/1535-7163.MCT-14-0862 25646013

[B150] Loganzo F. Sung M. Gerber H.-P. (2016). Mechanisms of resistance to antibody drug conjugates. Mol. Cancer Ther. 15 (12), 2825–2834. 10.1158/1535-7163.MCT-16-0408 27780876

[B151] Lucas A. T. Price L. S. L. Schorzman A. N. Storrie M. Piscitelli J. A. Razo J. (2018). Factors affecting the pharmacology of antibody-drug conjugates. Antibodies 7. 10.3390/antib7010010 31544862 PMC6698819

[B152] MacSween J. M. Warner N. L. Bankhurst A. D. Mackay I. R. (1972). Carcinoembryonic antigen in whole serum. Br. J. Cancer 26 (5), 356–360. 10.1038/bjc.1972.46 4117478 PMC2008638

[B153] Mahalingaiah P. K. Ciurlionis R. Durbin K. R. Yeager R. L. Philip B. K. Bawa B. (2019). Potential mechanisms of target-independent uptake and toxicity of antibody-drug conjugates. Pharmacol. and Ther. 200, 110–125. 10.1016/j.pharmthera.2019.04.008 31028836

[B154] Malik P. Phipps C. Edginton A. Blay J. (2017). Pharmacokinetic considerations for antibody-drug conjugates against cancer. Pharm. Res. 34 (12), 2579–2595. 10.1007/s11095-017-2259-3 28924691

[B155] Marchiò C. Annaratone L. Marques A. Casorzo L. Berrino E. Sapino A. (2021). Evolving concepts in HER2 evaluation in breast cancer: heterogeneity, HER2-low carcinomas and beyond. Seminars Cancer Biol. 72, 123–135. 10.1016/j.semcancer.2020.02.016 32112814

[B156] Markides D. M. Hita A. G. Merlin J. Reyes-Gibby C. Yeung S.-C. J. (2024). Antibody-drug conjugates: the toxicities and adverse effects that emergency physicians must know. Ann. Emerg. Med. 85 (3), 214–229. 10.1016/j.annemergmed.2024.10.015 39641680

[B157] Martin M. Fumoleau P. Dewar J. A. Albanell J. Limentani S. A. Campone M. (2016). Trastuzumab emtansine (T-DM1) plus docetaxel with or without pertuzumab in patients with HER2-positive locally advanced or metastatic breast cancer: results from a phase Ib/IIa study. Ann. Oncol. Official J. Eur. Soc. For Med. Oncol. 27 (7), 1249–1256. 10.1093/annonc/mdw157 27052654

[B158] Marusyk A. Janiszewska M. Polyak K. (2020). Intratumor heterogeneity: the rosetta stone of therapy resistance. Cancer Cell 37 (4), 471–484. 10.1016/j.ccell.2020.03.007 32289271 PMC7181408

[B159] Mathiot L. Baldini C. Letissier O. Hollebecque A. Bahleda R. Gazzah A. (2024). Exploring the role of target expression in treatment efficacy of antibody-drug conjugates (ADCs) in solid cancers: a comprehensive review. Curr. Oncol. Rep. 26 (10), 1236–1248. 10.1007/s11912-024-01576-9 39066847

[B160] Mazor Y. Oganesyan V. Yang C. Hansen A. Wang J. Liu H. (2015). Improving target cell specificity using a novel monovalent bispecific IgG design. MAbs 7 (2), 377–389. 10.1080/19420862.2015.1007816 25621507 PMC4622537

[B161] Mellman I. (1989). Organelles observed: lysosomes. Sci. (New York, N.Y.) 244 (4906), 853–854. 10.1126/science.244.4906.853 17802262

[B162] Miricescu D. Totan A. Stanescu-Spinu I.-I. Badoiu S. C. Stefani C. Greabu M. (2020). PI3K/AKT/mTOR signaling pathway in breast cancer: from molecular landscape to clinical aspects. Int. J. Mol. Sci. 22 (1), 173. 10.3390/ijms22010173 33375317 PMC7796017

[B163] Montero J. C. Del Carmen S. Abad M. Sayagués J. M. Barbáchano A. Fernández-Barral A. (2023). An amino acid transporter subunit as an antibody-drug conjugate target in colorectal cancer. J. Exp. and Clin. Cancer Res. CR 42 (1), 200. 10.1186/s13046-023-02784-0 37559159 PMC10410906

[B164] Moon S.-J. Govindan S. V. Cardillo T. M. D'Souza C. A. Hansen H. J. Goldenberg D. M. (2008). Antibody conjugates of 7-ethyl-10-hydroxycamptothecin (SN-38) for targeted cancer chemotherapy. J. Med. Chem. 51 (21), 6916–6926. 10.1021/jm800719t 18939816 PMC2661425

[B165] Moutafi M. Robbins C. J. Yaghoobi V. Fernandez A. I. Martinez-Morilla S. Xirou V. (2022). Quantitative measurement of HER2 expression to subclassify ERBB2 unamplified breast cancer. Laboratory Investigation; a J. Tech. Methods Pathology 102 (10), 1101–1108. 10.1038/s41374-022-00804-9 35595825

[B166] Mullard A. (2025). Dual-payload ADCs move into first oncology clinical trials. Nat. Rev. Drug Discov. 24 (8), 573–576. 10.1038/d41573-025-00121-y 40629007

[B167] Nakazawa Y. Miyano M. Tsukamoto S. Kogai H. Yamamoto A. Iso K. (2024). Delivery of a BET protein degrader *via* a CEACAM6-targeted antibody-drug conjugate inhibits tumour growth in pancreatic cancer models. Nat. Commun. 15 (1), 2192. 10.1038/s41467-024-46167-1 38467634 PMC10928091

[B168] Natsume A. Niwa R. Satoh M. (2009). Improving effector functions of antibodies for cancer treatment: enhancing ADCC and CDC. Drug Des. Dev. Ther. 3, 7–16. 19920917 PMC2769226

[B169] Ocana A. Vera-Badillo F. Seruga B. Templeton A. Pandiella A. Amir E. (2012). HER3 overexpression and survival in solid tumors: a meta-analysis. JNCI J. Natl. Cancer Inst. 105 (4), 266–273. 10.1093/jnci/djs501 23221996

[B170] Oflazoglu E. Stone I. J. Gordon K. Wood C. G. Repasky E. A. Grewal I. S. (2008). Potent anticarcinoma activity of the humanized anti-CD70 antibody h1F6 conjugated to the tubulin inhibitor auristatin *via* an uncleavable linker. Clin. Cancer Res. An Official J. Am. Assoc. For Cancer Res. 14 (19), 6171–6180. 10.1158/1078-0432.CCR-08-0916 18809969

[B171] Ogitani Y. Aida T. Hagihara K. Yamaguchi J. Ishii C. Harada N. (2016). DS-8201a, A novel HER2-Targeting ADC with a novel DNA topoisomerase I inhibitor, demonstrates a promising antitumor efficacy with differentiation from T-DM1. Clin. Cancer Res. An Official J. Am. Assoc. For Cancer Res. 22 (20), 5097–5108. 10.1158/1078-0432.CCR-15-2822 27026201

[B172] Oh D.-Y. Bang Y.-J. (2020). HER2-targeted therapies - a role beyond breast cancer. Nat. Rev. Clin. Oncol. 17 (1), 33–48. 10.1038/s41571-019-0268-3 31548601

[B173] Olayioye M. A. Neve R. M. Lane H. A. Hynes N. E. (2000). The ErbB signaling network: receptor heterodimerization in development and cancer. EMBO J. 19 (13), 3159–3167. 10.1093/emboj/19.13.3159 10880430 PMC313958

[B174] Patel H. Li J. Bo L. Mehta R. Ashby C. R. Wang S. (2024). Nanotechnology-based delivery systems to overcome drug resistance in cancer. Med. Rev. 4 (1), 5–30. 10.1515/mr-2023-0058 38515777 PMC10954245

[B175] Peng Z. Liu T. Wei J. Wang A. He Y. Yang L. (2021). Efficacy and safety of a novel anti-HER2 therapeutic antibody RC48 in patients with HER2-overexpressing, locally advanced or metastatic gastric or gastroesophageal junction cancer: a single-arm phase II study. Cancer Commun. Lond. Engl. 41 (11), 1173–1182. 10.1002/cac2.12214 34665942 PMC8626607

[B176] Petricoin E. F. Corgiat B. A. O’Shaughnessy J. LoRusso P. Weinberg K. Davis J. (2023). Abstract HER2-17: HER2-17 novel quantitative HER2 assay for determining dynamic HER2 expression in the HER2 IHC 0 “Ultra-Low” setting: implications for precision therapy in HER2- breast cancer. Cancer Res. 83 (5), HER2-17–17. 10.1158/1538-7445.Sabcs22-her2-17

[B177] Phillips G. D. L. Fields C. T. Li G. Dowbenko D. Schaefer G. Miller K. (2014). Dual targeting of HER2-positive cancer with trastuzumab emtansine and pertuzumab: critical role for neuregulin blockade in antitumor response to combination therapy. Clin. Cancer Res. An Official J. Am. Assoc. For Cancer Res. 20 (2), 456–468. 10.1158/1078-0432.CCR-13-0358 24097864

[B178] Pistilli B. Ibrahimi N. Lacroix-Triki M. D'Hondt V. Vicier C. Frenel J. S. (2023). 189O A phase II study of patritumab deruxtecan (HER3-DXd), in patients (pts) with advanced breast cancer (ABC), with biomarker analysis to characterize response to therapy (ICARUS-BREAST01). ESMO Open 8 (1), 101378. 10.1016/j.esmoop.2023.101378

[B179] Ponte J. F. Ab O. Lanieri L. Lee J. Coccia J. Bartle L. M. (2016). Mirvetuximab soravtansine (IMGN853), a folate receptor alpha-targeting antibody-drug conjugate, potentiates the activity of standard of care therapeutics in ovarian cancer models. Neoplasia (New York, N.Y.) 18 (12), 775–784. 10.1016/j.neo.2016.11.002 27889646 PMC5126132

[B180] Poreba M. (2020). Protease-activated prodrugs: strategies, challenges, and future directions. FEBS J. 287 (10), 1936–1969. 10.1111/febs.15227 31991521

[B181] Powell C. A. Camidge D. R. Modi S. Qin A. Taitt C. Lee C. (2020). 289P risk factors for interstitial lung disease in patients treated with trastuzumab deruxtecan from two interventional studies. Ann. Oncol. 31, S357–S358. 10.1016/j.annonc.2020.08.391

[B182] Powell C. A. Modi S. Iwata H. Takahashi S. Smit E. F. Siena S. (2022). Pooled analysis of drug-related interstitial lung disease and/or pneumonitis in nine trastuzumab deruxtecan monotherapy studies. ESMO Open 7 (4), 100554. 10.1016/j.esmoop.2022.100554 35963179 PMC9434416

[B183] Powles T. Rosenberg J. E. Sonpavde G. P. Loriot Y. Durán I. Lee J.-L. (2021). Enfortumab vedotin in previously treated advanced urothelial carcinoma. N. Engl. J. Med. 384 (12), 1125–1135. 10.1056/NEJMoa2035807 33577729 PMC8450892

[B184] Pretelli G. Mati K. Motta L. Stathis A. (2024). Antibody-drug conjugates combinations in cancer treatment. Explor Target Antitumor Ther. 5 (3), 714–741. 10.37349/etat.2024.00243 38966169 PMC11222717

[B185] Qiu M.-Z. Zhang Y. Guo Y. Guo W. Nian W. Liao W. (2022). Evaluation of safety of treatment with anti-epidermal growth factor receptor antibody drug conjugate MRG003 in patients with advanced solid tumors: a phase 1 nonrandomized clinical trial. JAMA Oncol. 8 (7), 1042–1046. 10.1001/jamaoncol.2022.0503 35511148 PMC9073657

[B186] Raab-Westphal S. Hart F. Sloot W. Shan M. Rasche N. Amendt C. (2024). Abstract 2362: preclinical efficacy and safety of M9140, a novel antibody-drug conjugate (ADC) with topoisomerase 1 (TOP1) inhibitor payload targeting carcinoembryonic antigen-related cell adhesion molecule 5 (CEACAM5)-expressing colorectal tumors. Cancer Res. 84 (6_Suppl. ment), 2362. 10.1158/1538-7445.Am2024-2362

[B187] Raghav K. Siena S. Takashima A. Kato T. Van den Eynde M. Pietrantonio F. (2024). Trastuzumab deruxtecan in patients with HER2-positive advanced colorectal cancer (DESTINY-CRC02): primary results from a multicentre, randomised, phase 2 trial. Lancet. Oncol. 25 (9), 1147–1162. 10.1016/S1470-2045(24)00380-2 39116902

[B188] Rathore M. Curry K. Huang W. Wright M. l. Martin D. Baek J. (2025). Leucine-Rich Alpha-2-Glycoprotein 1 promotes metastatic colorectal cancer growth through human epidermal growth factor receptor 3 signaling. Gastroenterology 168 (2), 300–315.e3. 10.1053/j.gastro.2024.10.004 39393543 PMC11769768

[B189] Saatci Ö. Borgoni S. Akbulut Ö. Durmuş S. Raza U. Eyüpoğlu E. (2018). Targeting PLK1 overcomes T-DM1 resistance *via* CDK1-dependent phosphorylation and inactivation of Bcl-2/xL in HER2-positive breast cancer. Oncogene 37 (17), 2251–2269. 10.1038/s41388-017-0108-9 29391599

[B190] Saleh M. N. Sugarman S. Murray J. Ostroff J. B. Healey D. Jones D. (2000). Phase I trial of the anti-lewis Y drug immunoconjugate BR96-doxorubicin in patients with lewis Y-expressing epithelial tumors. J. Clin. Oncol. Official J. Am. Soc. Clin. Oncol. 18 (11), 2282–2292. 10.1200/JCO.2000.18.11.2282 10829049

[B191] Samantasinghar A. Sunildutt N. P. Ahmed F. Soomro A. M. Salih A. R. C. Parihar P. (2023). A comprehensive review of key factors affecting the efficacy of antibody drug conjugate. Biomed. and Pharmacother. = Biomedecine and Pharmacother. 161, 114408. 10.1016/j.biopha.2023.114408 36841027

[B192] Sartore-Bianchi A. Lonardi S. Martino C. Fenocchio E. Tosi F. Ghezzi S. (2020). Pertuzumab and trastuzumab emtansine in patients with HER2-amplified metastatic colorectal cancer: the phase II HERACLES-B trial. ESMO Open 5 (5), e000911. 10.1136/esmoopen-2020-000911 32988996 PMC7523198

[B193] Sawant M. S. Streu C. N. Wu L. Tessier P. M. (2020). Toward drug-like multispecific antibodies by design. Int. J. Mol. Sci. 21 (20). 10.3390/ijms21207496 33053650 PMC7589779

[B194] Schettini F. Barbao P. Brasó-Maristany F. Galván P. Martínez D. Paré L. (2021). Identification of cell surface targets for CAR-T cell therapies and antibody-drug conjugates in breast cancer. ESMO Open 6 (3), 100102. 10.1016/j.esmoop.2021.100102 33838601 PMC8038941

[B195] Scribner J. A. Hicks S. W. Sinkevicius K. W. Yoder N. C. Diedrich G. Brown J. G. (2022). Preclinical evaluation of IMGC936, a next-generation maytansinoid-based antibody-drug conjugate targeting ADAM9-expressing tumors. Mol. Cancer Ther. 21 (7), 1047–1059. 10.1158/1535-7163.MCT-21-0915 35511740

[B196] Segal N. H. Dotan E. Berlin J. D. Starodub A. N. Guarino M. J. Saltz L. B. (2014). Abstract CT211: IMMU-130, an SN-38 antibody-drug conjugate (ADC) targeting CEACAM5, is therapeutically active in metastatic colorectal cancer (mCRC): initial clinical results of two phase I studies. Cancer Res. 74 (19_Suppl. ment), CT211. 10.1158/1538-7445.Am2014-ct211

[B197] Sellmann C. Doerner A. Knuehl C. Rasche N. Sood V. Krah S. (2016). Balancing selectivity and efficacy of bispecific epidermal growth factor receptor (EGFR) × c-MET antibodies and antibody-drug conjugates. J. Biol. Chem. 291 (48), 25106–25119. 10.1074/jbc.M116.753491 27694443 PMC5122778

[B198] Sheng X. Yan X. Wang L. Shi Y. Yao X. Luo H. (2021). Open-label, multicenter, phase II study of RC48-ADC, a HER2-Targeting antibody-drug conjugate, in patients with locally advanced or metastatic urothelial carcinoma. Clin. Cancer Res. An Official J. Am. Assoc. For Cancer Res. 27 (1), 43–51. 10.1158/1078-0432.CCR-20-2488 33109737

[B199] Sheng X. Wang L. He Z. Shi Y. Luo H. Han W. (2024). Efficacy and safety of disitamab vedotin in patients with human epidermal growth factor receptor 2-Positive locally advanced or metastatic urothelial carcinoma: a combined analysis of two phase II clinical trials. J. Clin. Oncol. Official J. Am. Soc. Clin. Oncol. 42 (12), 1391–1402. 10.1200/JCO.22.02912 37988648 PMC11095880

[B200] Sheyi R. de la Torre B. G. Albericio F. (2022). Linkers: an assurance for controlled delivery of antibody-drug conjugate. Pharmaceutics 14 (2), 396. 10.3390/pharmaceutics14020396 35214128 PMC8874516

[B201] Shi J. Sun Z. Gao Z. Huang D. Hong H. Gu J. (2023). Radioimmunotherapy in colorectal cancer treatment: present and future. Front. Immunol. 14, 1105180. 10.3389/fimmu.2023.1105180 37234164 PMC10206275

[B202] Shimozaki K. Fukuoka S. Ooki A. Yamaguchi K. (2024). HER2-low gastric cancer: is the subgroup targetable? ESMO Open 9 (9), 103679. 10.1016/j.esmoop.2024.103679 39178538 PMC11386020

[B203] Siena S. Di Bartolomeo M. Raghav K. Masuishi T. Loupakis F. Kawakami H. (2021). Trastuzumab deruxtecan (DS-8201) in patients with HER2-expressing metastatic colorectal cancer (DESTINY-CRC01): a multicentre, open-label, phase 2 trial. Lancet. Oncol. 22 (6), 779–789. 10.1016/S1470-2045(21)00086-3 33961795

[B204] Sierke S. L. Cheng K. Kim H. H. Koland J. G. (1997). Biochemical characterization of the protein tyrosine kinase homology domain of the ErbB3 (HER3) receptor protein. Biochem. J. 322 (Pt 3), 757–763. 10.1042/bj3220757 9148746 PMC1218252

[B205] Singh A. P. Guo L. Verma A. Wong G.G.-L. Thurber G. M. Shah D. K. (2020). Antibody coadministration as a strategy to overcome binding-site barrier for ADCs: a quantitative investigation. AAPS J. 22 (2), 28. 10.1208/s12248-019-0387-x 31938899 PMC8382310

[B206] Siravegna G. Mussolin B. Buscarino M. Corti G. Cassingena A. Crisafulli G. (2015). Clonal evolution and resistance to EGFR blockade in the blood of colorectal cancer patients. Nat. Med. 21 (7), 795–801. 10.1038/nm.3870 26030179 PMC4868598

[B207] Sobhani N. D'Angelo A. Pittacolo M. Mondani G. Generali D. (2024). Future AI will Most likely predict antibody-drug conjugate response in oncology: a review and expert opinion. Cancers (Basel) 16 (17). 10.3390/cancers16173089 PMC11394064 39272947

[B208] Spitzmüller A. Kapil A. Shumilov A. Chan J. Konstantinidou L. Hassan Z. (2023). Abstract P6-04-03: computational pathology based HER2 expression quantification in HER2-low breast cancer. Cancer Res. 83 (5_Suppl. ment), P6. 10.1158/1538-7445.Sabcs22-p6-04-03

[B209] Staudacher A. H. Brown M. P. (2017). Antibody drug conjugates and bystander killing: is antigen-dependent internalisation required? Br. J. Cancer 117 (12), 1736–1742. 10.1038/bjc.2017.367 29065110 PMC5729478

[B210] Strebhardt K. Ullrich A. (2008). Paul Ehrlich's magic bullet concept: 100 years of progress. Nat. Rev. Cancer 8 (6), 473–480. 10.1038/nrc2394 18469827

[B211] Su C.-C. (2018). Tanshinone IIA inhibits gastric carcinoma AGS cells by decreasing the protein expression of VEGFR and blocking Ras/Raf/MEK/ERK pathway. Int. J. Mol. Med. 41 (4), 2389–2396. 10.3892/ijmm.2018.3407 29393362

[B212] Su Z. Xiao D. Xie F. Liu L. Wang Y. Fan S. (2021). Antibody-drug conjugates: recent advances in linker chemistry. Acta Pharm. Sin. B 11 (12), 3889–3907. 10.1016/j.apsb.2021.03.042 35024314 PMC8727783

[B213] Sun X. Ponte J. F. Yoder N. C. Laleau R. Coccia J. Lanieri L. (2017). Effects of drug-antibody ratio on pharmacokinetics, biodistribution, efficacy, and tolerability of antibody-maytansinoid conjugates. Bioconjugate Chem. 28 (5), 1371–1381. 10.1021/acs.bioconjchem.7b00062 28388844

[B214] Sung M. S. Tan X. Hosselet C. Cinque M. Upeslacis E. Golas J. (2016). Abstract 2113: caveolae-mediated endocytosis as a novel mechanism of resistance to T-DM1 ADC. Cancer Res. 76 (14_Suppl. ment), 2113. 10.1158/1538-7445.Am2016-2113

[B215] Sung M. Tan X. Lu B. Golas J. Hosselet C. Wang F. (2018). Caveolae-mediated endocytosis as a novel mechanism of resistance to trastuzumab emtansine (T-DM1). Mol. Cancer Ther. 17 (1), 243–253. 10.1158/1535-7163.MCT-17-0403 29054985

[B216] Sung H. Ferlay J. Siegel R. L. Laversanne M. Soerjomataram I. Jemal A. (2021). Global cancer statistics 2020: GLOBOCAN estimates of incidence and mortality worldwide for 36 cancers in 185 countries. CA A Cancer J. For Clin. 71 (3), 209–249. 10.3322/caac.21660 33538338

[B217] Suwaidan A. A. Lau D. K. Chau I. (2022). HER2 targeted therapy in colorectal cancer: new horizons. Cancer Treat. Rev. 105, 102363. 10.1016/j.ctrv.2022.102363 35228040

[B218] Suzuki Y. Ng S. B. Chua C. Leow W. Q. Chng J. Liu S. Y. (2017). Multiregion ultra-deep sequencing reveals early intermixing and variable levels of intratumoral heterogeneity in colorectal cancer. Mol. Oncol. 11 (2), 124–139. 10.1002/1878-0261.12012 28145097 PMC5527459

[B219] Tabariès S. Robert A. Marcil A. Ling B. Acchione M. Lippens J. (2024). Anti-Claudin-2 antibody-drug conjugates for the treatment of colorectal cancer liver metastasis. Mol. Cancer Ther. 23 (10), 1459–1470. 10.1158/1535-7163.MCT-23-0393 38902871

[B220] Takegawa N. Nonagase Y. Yonesaka K. Sakai K. Maenishi O. Ogitani Y. (2017). DS-8201a, a new HER2-targeting antibody-drug conjugate incorporating a novel DNA topoisomerase I inhibitor, overcomes HER2-positive gastric cancer T-DM1 resistance. Int. J. Cancer 141 (8), 1682–1689. 10.1002/ijc.30870 28677116

[B221] Takegawa N. Tsurutani J. Kawakami H. Yonesaka K. Kato R. Haratani K. (2019). [fam-] trastuzumab deruxtecan, antitumor activity is dependent on HER2 expression level rather than on HER2 amplification. Int. J. Cancer 145 (12), 3414–3424. 10.1002/ijc.32408 31087550

[B222] Tan G. Lin C. Huang C. Chen B. Chen J. Shi Y. (2022). Radiosensitivity of colorectal cancer and radiation-induced gut damages are regulated by gasdermin E. Cancer Lett. 529, 1–10. 10.1016/j.canlet.2021.12.034 34979164

[B223] Tan H. N. Morcillo M. A. Lopez J. Minchom A. Sharp A. Paschalis A. (2025). Treatment-related adverse events of antibody drug-conjugates in clinical trials. J. Hematol. Oncol. 18 (1), 71. 10.1186/s13045-025-01720-3 40611310 PMC12231679

[B224] Tang H. Liu Y. Yu Z. Sun M. Lin L. Liu W. (2019). The analysis of key factors related to ADCs structural design. Front. Pharmacol. 10, 10–2019. 10.3389/fphar.2019.00373 31068807 PMC6491742

[B225] Tang M. Garg A. Bonate P. L. Rosenberg J. E. Matsangou M. Kadokura T. (2024a). Clinical pharmacology of the antibody-drug conjugate enfortumab vedotin in advanced urothelial carcinoma and other malignant solid tumors. Clin. Pharmacokinet. 63 (4), 423–438. 10.1007/s40262-024-01369-0 38609704 PMC11052883

[B226] Tang S. C. Wynn C. Le T. McCandless M. Zhang Y. Patel R. (2024b). Influence of antibody-drug conjugate cleavability, drug-to-antibody ratio, and free payload concentration on systemic toxicities: a systematic review and meta-analysis. Cancer Metastasis Rev. 44 (1), 18. 10.1007/s10555-024-10231-5 39704752 PMC11662062

[B227] Tarantino P. Tolaney S. M. (2022). The dawn of the antibody-drug conjugates era: how T-DM1 reinvented the future of chemotherapy for solid tumors. Cancer Res. 82 (20), 3659–3661. 10.1158/0008-5472.CAN-22-2324 36245247

[B228] Tarantino P. Modi S. Tolaney S. M. Cortés J. Hamilton E. P. Kim S.-B. (2021). Interstitial lung disease induced by Anti-ERBB2 antibody-drug conjugates: a review. JAMA Oncol. 7 (12), 1873–1881. 10.1001/jamaoncol.2021.3595 34647966

[B229] Tarantino P. Carmagnani Pestana R. Corti C. Modi S. Bardia A. Tolaney S. M. (2022). Antibody-drug conjugates: smart chemotherapy delivery across tumor histologies. CA A Cancer J. For Clin. 72 (2), 165–182. 10.3322/caac.21705 34767258

[B230] Tiernan J. P. Perry S. L. Verghese E. T. West N. P. Yeluri S. Jayne D. G. (2013). Carcinoembryonic antigen is the preferred biomarker for *in vivo* colorectal cancer targeting. Br. J. Cancer 108 (3), 662–667. 10.1038/bjc.2012.605 23322207 PMC3593555

[B231] Tsuchikama K. Anami Y. Ha S. Y. Y. Yamazaki C. M. (2024). Exploring the next generation of antibody-drug conjugates. Nat. Rev. Clin. Oncol. 21 (3), 203–223. 10.1038/s41571-023-00850-2 38191923

[B232] Tsumura R. Manabe S. Takashima H. Koga Y. Yasunaga M. Matsumura Y. (2018). Influence of the dissociation rate constant on the intra-tumor distribution of antibody-drug conjugate against tissue factor. J. Control. Release Official J. Control. Release Soc. 284, 49–56. 10.1016/j.jconrel.2018.06.016 29906553

[B233] Tsurutani J. Iwata H. Krop I. Jänne P. A. Doi T. Takahashi S. (2020). Targeting HER2 with trastuzumab deruxtecan: a dose-expansion, phase I study in multiple advanced solid tumors. Cancer Discov. 10 (5), 688–701. 10.1158/2159-8290.CD-19-1014 32213540 PMC8292921

[B234] Urbanski R. Carrithers S. L. Waldman S. A. (1995). Internalization of *E. coli* ST mediated by guanylyl cyclase C in T84 human colon carcinoma cells. Biochimica Biophysica Acta 1245 (1), 29–36. 10.1016/0304-4165(95)00068-m 7654763

[B235] Vaghi C. Tosi F. Mauri G. Bonazzina E. Amatu A. Bencardino K. (2025). Targeting HER2 in metastatic colorectal cancer: current therapies, biomarker refinement, and emerging strategies. Drugs 86, 37–57. 10.1007/s40265-025-02253-2 41247589 PMC12799626

[B236] Van Cutsem E. Lenz H.-J. Köhne C.-H. Heinemann V. Tejpar S. Melezínek I. (2015). Fluorouracil, leucovorin, and irinotecan plus cetuximab treatment and RAS mutations in colorectal cancer. J. Clin. Oncol. Official J. Am. Soc. Clin. Oncol. 33 (7), 692–700. 10.1200/JCO.2014.59.4812 25605843

[B237] van den Bent M. Gan H. K. Lassman A. B. Kumthekar P. Merrell R. Butowski N. (2017). Efficacy of depatuxizumab mafodotin (ABT-414) monotherapy in patients with EGFR-amplified, recurrent glioblastoma: results from a multi-center, international study. Cancer Chemother. Pharmacol. 80 (6), 1209–1217. 10.1007/s00280-017-3451-1 29075855 PMC5686264

[B238] Villacampa G. Cresta Morgado P. Carità L. Navarro V. Pascual T. Dienstmann R. (2024). Safety and efficacy of antibody-drug conjugates plus immunotherapy in solid tumours: a systematic review and meta-analysis. Cancer Treat. Rev. 131, 102847. 10.1016/j.ctrv.2024.102847 39454548

[B239] Wagle N. S. Nogueira L. Devasia T. P. Mariotto A. B. Yabroff K. R. Islami F. (2025). Cancer treatment and survivorship statistics, 2025. CA Cancer J. Clin. 75 (4), 308–340. 10.3322/caac.70011 40445120 PMC12223361

[B240] Wahl A. F. Donaldson K. L. Mixan B. J. Trail P. A. Siegall C. B. (2001). Selective tumor sensitization to taxanes with the mAb-drug conjugate cBR96-doxorubicin. Int. J. Cancer 93 (4), 590–600. 10.1002/ijc.1364 11477565

[B241] Waldman S. A. Camilleri M. (2018). Guanylate cyclase-C as a therapeutic target in gastrointestinal disorders. Gut 67 (8), 1543–1552. 10.1136/gutjnl-2018-316029 29563144 PMC6204952

[B242] Waldman S. A. Barber M. Pearlman J. Park J. George R. Parkinson S. J. (1998). Heterogeneity of guanylyl cyclase C expressed by human colorectal cancer cell lines *in vitro* . Cancer Epidemiol. Biomarkers and Prev. A Publ. Am. Assoc. For Cancer Res. Cosponsored by Am. Soc. Prev. Oncol. 7 (6), 505–514. 9641495

[B243] Wang H. Wang W. Xu Y. Yang Y. Chen X. Quan H. (2017). Aberrant intracellular metabolism of T-DM1 confers T-DM1 resistance in human epidermal growth factor receptor 2-positive gastric cancer cells. Cancer Sci. 108 (7), 1458–1468. 10.1111/cas.13253 28388007 PMC5497802

[B244] Wang R. Lai Q. Tang L. Tao Y. Yao Y. Liu Y. (2018). A novel 5T4-targeting antibody-drug conjugate H6-DM4 exhibits potent therapeutic efficacy in gastrointestinal tumor xenograft models. Am. J. Cancer Res. 8 (4), 610–623. 29736307 PMC5934552

[B245] Wang R. Fang P. Chen X. Ji J. Yu D. Mei F. (2025a). Overcoming multidrug resistance in gastrointestinal cancers with a CDH17-targeted ADC conjugated to a DNA topoisomerase inhibitor. Cell Rep. Med. 6 (7), 102213. 10.1016/j.xcrm.2025.102213 40602407 PMC12281427

[B246] Wang R. Hu B. Pan Z. Mo C. Zhao X. Liu G. (2025b). Antibody-drug conjugates (ADCs): current and future biopharmaceuticals. J. Hematol. Oncol., 1756–8722. 10.1186/s13045-025-01704-3 PMC12044742 40307936

[B247] Wei Q. Li P. Yang T. Zhu J. Sun L. Zhang Z. (2024). The promise and challenges of combination therapies with antibody-drug conjugates in solid tumors. J. Hematol. and Oncol. 17 (1), 1. 10.1186/s13045-023-01509-2 38178200 PMC10768262

[B248] Weng W. Meng T. Pu J. Ma L. Shen Y. Wang Z. (2023a). AMT-562, a novel HER3-targeting antibody-drug conjugate, demonstrates a potential to broaden therapeutic opportunities for HER3-expressing tumors. Mol. Cancer Ther. 22 (9), 1013–1027. 10.1158/1535-7163.MCT-23-0198 37302522 PMC10477830

[B249] Weng W. Meng T. Zhao Q. Shen Y. Fu G. Shi J. (2023b). Antibody-exatecan conjugates with a novel self-immolative moiety overcome resistance in Colon and lung cancer. Cancer Discov. 13 (4), 950–973. 10.1158/2159-8290.CD-22-1368 36693125

[B250] Williams M. Spreafico A. Vashisht K. Hinrichs M. J. (2020). Patient selection strategies to maximize therapeutic index of antibody-drug conjugates: prior approaches and future directions. Mol. Cancer Ther. 19 (9), 1770–1783. 10.1158/1535-7163.MCT-19-0993 32546659

[B251] Wong W. H. M. Ng R. Aziz M. I. A. Ong B.S.-K. Ng K. (2025). Cost-effectiveness analysis of trastuzumab emtansine for second-line treatment of HER2+ advanced breast cancer in Singapore. Expert Rev. Pharmacoeconomics and Outcomes Res. 25 (5), 679–687. 10.1080/14737167.2025.2456065 39819168

[B252] Wu Y. Ginther C. Kim J. Mosher N. Chung S. Slamon D. (2012). Expression of Wnt3 activates Wnt/β-catenin pathway and promotes EMT-like phenotype in trastuzumab-resistant HER2-overexpressing breast cancer cells. Mol. Cancer Res. MCR 10 (12), 1597–1606. 10.1158/1541-7786.MCR-12-0155-T 23071104 PMC3732195

[B253] Wu S. Zhang Q. Zhang F. Meng F. Liu S. Zhou R. (2019). HER2 recruits AKT1 to disrupt STING signalling and suppress antiviral defence and antitumour immunity. Nat. Cell Biol. 21 (8), 1027–1040. 10.1038/s41556-019-0352-z 31332347

[B254] Wu X. Xu L. Li X. Zhou Y. Han X. Zhang W. (2023a). A HER2-targeting antibody-MMAE conjugate RC48 sensitizes immunotherapy in HER2-positive colon cancer by triggering the cGAS-STING pathway. Cell Death and Dis. 14 (8), 550. 10.1038/s41419-023-06073-8 37620320 PMC10449775

[B255] Wu Y. Li W. Chen X. Wang H. Su S. Xu Y. (2023b). DOG1 as a novel antibody-drug conjugate target for the treatment of multiple gastrointestinal tumors and liver metastasis. Front. Immunol. 14, 1051506. 10.3389/fimmu.2023.1051506 36776873 PMC9909470

[B256] Wu J. Li Q. Cheng X. Dai C. Huang Q. Wang Y. (2025). RC48 induces senescence in HER2-Expressing Colon cancer cells by activating the CDKN1A-RB-E2F1 pathway. Curr. Cancer Drug Targets. 10.2174/0115680096388633250704101500 40671237

[B257] Xiao D. Liu L. Xie F. Dong J. Wang Y. Xu X. (2024). Azobenzene-based linker strategy for selective activation of antibody-drug conjugates. Angewandte Chemie Int. Ed. Engl. 63 (16), e202310318. 10.1002/anie.202310318 38369681

[B258] Xie Y.-H. Chen Y.-X. Fang J.-Y. (2020). Comprehensive review of targeted therapy for colorectal cancer. Signal Transduct. Target. Ther. 5 (1), 22. 10.1038/s41392-020-0116-z 32296018 PMC7082344

[B259] Xu S. (2015). Internalization, trafficking, intracellular processing and actions of antibody-drug conjugates. Pharm. Res. 32 (11), 3577–3583. 10.1007/s11095-015-1729-8 26108878

[B260] Yaeger R. Chatila W. K. Lipsyc M. D. Hechtman J. F. Cercek A. Sanchez-Vega F. (2018). Clinical sequencing defines the genomic landscape of metastatic colorectal cancer. Cancer Cell 33 (1), 125–136. 10.1016/j.ccell.2017.12.004 29316426 PMC5765991

[B261] Yao X. Jiang J. Wang X. Huang C. Li D. Xie K. (2015). A novel humanized anti-HER2 antibody conjugated with MMAE exerts potent anti-tumor activity. Breast Cancer Res. Treat. 153 (1), 123–133. 10.1007/s10549-015-3503-3 26253944

[B262] Yao H. Yan M. Tong Z. Wu X. Ryu M.-H. Park J. J. (2024). Safety, efficacy, and pharmacokinetics of SHR-A1811, a human epidermal growth factor receptor 2-Directed antibody-drug conjugate, in human epidermal growth factor receptor 2-Expressing or mutated advanced solid tumors: a global phase I trial. J. Clin. Oncol. Official J. Am. Soc. Clin. Oncol. 42 (29), 3453–3465. 10.1200/JCO.23.02044 38900984

[B263] Yarden Y. Sliwkowski M. X. (2001). Untangling the ErbB signalling network. Nat. Rev. Mol. Cell Biol. 2 (2), 127–137. 10.1038/35052073 11252954

[B264] Yardley D. A. (2013). Drug resistance and the role of combination chemotherapy in improving patient outcomes. Int. J. Breast Cancer 2013, 137414. 10.1155/2013/137414 23864953 PMC3707274

[B265] Ye Z. Zhang Y. Liu Y. Liu Y. Tu J. Shen Y. (2021). EGFR targeted cetuximab-valine-citrulline (vc)-doxorubicin Immunoconjugates- loaded bovine serum albumin (BSA) nanoparticles for colorectal tumor therapy. Int. J. Nanomedicine 16, 2443–2459. 10.2147/IJN.S289228 33814909 PMC8009551

[B266] Yoder N. C. Bai C. Tavares D. Widdison W. C. Whiteman K. R. Wilhelm A. (2019). A case study comparing heterogeneous Lysine- and site-specific cysteine-conjugated maytansinoid antibody-drug conjugates (ADCs) illustrates the benefits of lysine conjugation. Mol. Pharm. 16 (9), 3926–3937. 10.1021/acs.molpharmaceut.9b00529 31287952

[B267] Yonesaka K. Takegawa N. Watanabe S. Haratani K. Kawakami H. Sakai K. (2019). An HER3-targeting antibody-drug conjugate incorporating a DNA topoisomerase I inhibitor U3-1402 conquers EGFR tyrosine kinase inhibitor-resistant NSCLC. Oncogene 38 (9), 1398–1409. 10.1038/s41388-018-0517-4 30302022

[B268] Yoshino T. Di Bartolomeo M. Raghav K. Masuishi T. Loupakis F. Kawakami H. (2023). Final results of DESTINY-CRC01 investigating trastuzumab deruxtecan in patients with HER2-expressing metastatic colorectal cancer. Nat. Commun. 14 (1), 3332. 10.1038/s41467-023-38032-4 37286557 PMC10247780

[B269] Yu M. Ocana A. Tannock I. F. (2013). Reversal of ATP-binding cassette drug transporter activity to modulate chemoresistance: why has it failed to provide clinical benefit? Cancer Metastasis Rev. 32 (1-2), 211–227. 10.1007/s10555-012-9402-8 23093326

[B270] Yu S.-F. Zheng B. Go M. Lau J. Spencer S. Raab H. (2015). A novel Anti-CD22 anthracycline-based antibody-drug conjugate (ADC) that overcomes resistance to auristatin-based ADCs. Clin. Cancer Res. An Official J. Am. Assoc. For Cancer Res. 21 (14), 3298–3306. 10.1158/1078-0432.CCR-14-2035 25840969

[B271] Yu J. Song Y. Tian W. (2020). How to select IgG subclasses in developing anti-tumor therapeutic antibodies. J. Hematol. and Oncol. 13 (1), 45. 10.1186/s13045-020-00876-4 32370812 PMC7201658

[B272] Yu B. Kang J. Lei H. Li Z. Yang H. Zhang M. (2024). Immunotherapy for colorectal cancer. Front. Immunol. 15, 15–2024. 10.3389/fimmu.2024.1433315 39238638 PMC11375682

[B273] Zhang N. Zeng Y. Du W. Zhu J. Shen D. Liu Z. (2016). The EGFR pathway is involved in the regulation of PD-L1 expression *via* the IL-6/JAK/STAT3 signaling pathway in EGFR-mutated non-small cell lung cancer. Int. J. Oncol. 49 (4), 1360–1368. 10.3892/ijo.2016.3632 27499357

[B274] Zhang S. Zhou D. Zheng C. Xiong P. Zhu W. Zheng D. (2021). Preclinical evaluation of a novel antibody-drug conjugate targeting DR5 for lymphoblastic leukemia therapy. Mol. Ther. Oncolytics 21, 329–339. 10.1016/j.omto.2021.04.013 34141870 PMC8173093

[B275] Zhang H. Sun J. Zhang Y. Zhang Z. Wang X. Liu Z. (2023). Preparation of an Ultrahigh-DAR PDL1 monoclonal antibody-polymeric-SN38 conjugate for precise colon cancer therapy. Biomaterials 301, 122285. 10.1016/j.biomaterials.2023.122285 37619265

[B276] Zhang T. Xu J. Yin J. Gao Y. Zheng H. Fu B. (2025). SHR-A1811, a novel anti-HER2 antibody-drug conjugate with optimal drug-to-antibody ratio, efficient tumor killing potency, and favorable safety profiles. PloS One 20 (6), e0326691. 10.1371/journal.pone.0326691 40569929 PMC12200682

[B277] Zhao P. Zhang Y. Li W. Jeanty C. Xiang G. Dong Y. (2020). Recent advances of antibody drug conjugates for clinical applications. Acta Pharm. Sin. B 10 (9), 1589–1600. 10.1016/j.apsb.2020.04.012 33088681 PMC7564033

[B278] Zheng C. Zhou D. Li W. Duan Y. Xu M. Liu J. (2023). Therapeutic efficacy of a MMAE-based anti-DR5 drug conjugate Oba01 in preclinical models of pancreatic cancer. Cell Death and Dis. 14 (4), 295. 10.1038/s41419-023-05820-1 37120688 PMC10148860

[B279] Zhou D. Tang E. j. Wang W. Xiao Y. Huang J. Liu J. (2025a). Combined therapy with DR5-targeting antibody-drug conjugate and CDK inhibitors as a strategy for advanced colorectal cancer. Cell Rep. Med. 6 (6), 102158. 10.1016/j.xcrm.2025.102158 40449480 PMC12208337

[B280] Zhou L. Yang K. W. Zhang S. Yan X. Q. Li S. M. Xu H. Y. (2025b). Disitamab vedotin plus toripalimab in patients with locally advanced or metastatic urothelial carcinoma (RC48-C014): a phase Ib/II dose-escalation and dose-expansion study. Ann. Oncol. Official J. Eur. Soc. For Med. Oncol. 36 (3), 331–339. 10.1016/j.annonc.2024.12.002 39662628

[B281] Zhu X. Huo S. Xue C. An B. Qu J. (2020). Current LC-MS-based strategies for characterization and quantification of antibody-drug conjugates. J. Pharm. Analysis 10 (3), 209–220. 10.1016/j.jpha.2020.05.008 32612867 PMC7322744

[B282] Zhu Y. Liu K. Wang K. Zhu H. (2023). Treatment-related adverse events of antibody-drug conjugates in clinical trials: a systematic review and meta-analysis. Cancer 129 (2), 283–295. 10.1002/cncr.34507 36408673 PMC10099922

